# Effective degradation of synthetic micropollutants and real textile wastewater via a visible light-activated persulfate system using novel spinach leaf-derived biochar

**DOI:** 10.1007/s11356-024-32829-6

**Published:** 2024-03-11

**Authors:** Mohamed Mohamed Gaber, Mahmoud Samy, Hassan Shokry

**Affiliations:** 1 Environmental Engineering Department, Egypt-Japan University of Science and Technology (E-JUST), New Borg El-Arab City 21934, Alexandria, Egypt; 2 Public Works Engineering Department, Faculty of Engineering, Mansoura University, Mansoura, 35516 Egypt

**Keywords:** Biochar, Degradation mechanism, Methylene blue, Persulfate activation, Real wastewater, Spinach leaves, Visible light

## Abstract

A novel biochar (BC), derived from spinach leaves, was utilized as an activator for persulfate (PS) in the degradation of methylene blue (MB) dye under visible light conditions. Thorough analyses were conducted to characterize the physical and chemical properties of the biochar. The (BC + light)/PS system exhibited superior MB degradation efficiency at 83.36%, surpassing the performance of (BC + light)/hydrogen peroxide and (BC + light)/peroxymonosulfate systems. The optimal conditions were ascertained through the implementation of response surface methodology. Moreover, the (BC + light)/PS system demonstrated notable degradation ratios of 90.82%, 81.88%, and 84.82% for bromothymol blue dye, paracetamol, and chlorpyrifos, respectively, under optimal conditions. The predominant reactive species responsible for MB degradation were identified as sulfate radicals. Notably, the proposed system consistently achieved high removal efficiencies of 99.02%, 96.97%, 94.94%, 92%, and 90.35% for MB in five consecutive runs. The applicability of the suggested system was further validated through its effectiveness in treating real textile wastewater, exhibiting a substantial MB removal efficiency of 98.31% and dissolved organic carbon mineralization of 87.49%.

**Supplementary Information:**

The online version contains supplementary material available at 10.1007/s11356-024-32829-6.

## Introduction

Methylene blue (MB) stands out as a prominent cationic organic dye extensively employed as a coloring agent in diverse industries such as textiles, printing, papermaking, cotton dyeing, and leather (Liang et al. 2021). Renowned for its elevated toxicity and potential carcinogenicity, MB has been implicated in various diseases affecting both humans and animals (Al-Tohamy et al. 2022), contributing to a spectrum of health issues ranging from dermatitis to central nervous system disorders (Ji et al. 2020). Traditional biological methods for MB treatment encounter challenges due to its low biodegradability (Kumar et al. 2020). Furthermore, conventional chemical and physical treatment techniques often prove expensive and generate sludge as a byproduct, necessitating additional treatment steps (Teixeira et al. 2022). Consequently, there is a pressing need to develop innovative technologies capable of more efficiently removing MB from water and wastewater.

Advanced oxidation processes (AOPs) have emerged as a promising strategy for effectively addressing persistent organic pollutants (Darwish et al. 2022). Within AOPs, sulfate radicals exhibit distinct advantages, featuring a longer half-life of 30–40 µs compared to the less than 1 µs half-life of hydroxyl radicals (Nie et al. 2023). Additionally, sulfate radicals possess a higher redox potential (2.5–3.2 V) in contrast to hydroxyl radicals (1.8–2.7 V) (Zhang et al. 2022b). Notably, sulfate radicals demonstrate operational efficiency across a broader pH range, spanning from 3 to 10 (El-Bestawy et al. 2023). Furthermore, sulfate radicals facilitate the rapid and non-selective oxidation of organic pollutants into smaller organic molecules, carbon dioxide, and water (Habibi et al. 2022; Li et al. 2022a). The generation of sulfate radicals can be achieved through the activation of peroxymonosulfate (PMS, SO_5_^–2^) or peroxydisulfate (PS, S_2_O_8_^–2^) anions (Cornejo et al. 2023a). Various physical methods, including solar energy, heating, ultraviolet (UV) irradiation, or chemical methods utilizing bases, phenols, quinones, and transition metals, can be employed for PS activation (Cornejo et al. 2023b).

The utilization of UV light in PS activation presents notable limitations, with UV light constituting only 4% of the total sunlight spectrum, thereby severely constraining the practicality of harnessing solar energy for such processes (Song et al. 2022). Moreover, human overexposure to UV radiation, in the absence of proper protection, heightens the risk of severe health issues, including blinding eye diseases, premature skin aging, and skin cancer (Zhang et al. 2023). Therefore, the appeal of employing cost-effective visible light for PS activation is evident, offering significant potential for decontaminating MB while harnessing a considerably larger portion (48%) of the solar spectrum without the associated health risks (Deng et al. 2022).

Biochar (BC) stands out as an economical material characterized by a high carbon content and abundant hydroxy and carboxyl groups, contributing to its porous structure (Li et al. 2023a). The presence of functional groups in BC enables it to effectively activate PS and serve as a catalyst in the degradation of organic pollutants (Xu et al. 2022). Utilizing BC in PS activation systems offers a solution to challenges associated with high energy input and metal leaching, thereby enhancing the feasibility of BC-activated PS applications (Yadav et al. 2021). Given the substantial daily production of food waste, efficient management becomes imperative to mitigate environmental harm from landfills. In this context, BC emerges as a sustainable waste management solution, presenting a more environmentally friendly alternative to conventional techniques like landfilling or incineration (Yu et al. 2022).

In the conducted study, a BC system coupled with visible light was implemented to activate PS and facilitate the degradation of MB dye. The BC utilized in this experiment was derived from spinach leaves. A comprehensive investigation was conducted to assess the impact of various operational parameters, including pH, initial pollutant concentration, catalyst dosage, and PS concentration. Additionally, the study delved into the reusability and stability of the synthesized BC catalyst. The efficiency of the system was evaluated across different water matrices and a diverse range of organic pollutants. Furthermore, scavenging experiments were employed to elucidate the degradation mechanism, and the mineralization potential of actual wastewater sourced from a textile company was explored.

## Materials and methods

### Materials and chemicals

Real textile wastewater samples were generously provided by a local textile company located in Borg El-Arab City, Alexandria, Egypt. Additionally, samples of both lake water (LW) and sea water (SW) were gathered from Alexandria’s Mediterranean Coast and Lake Mariout, respectively. Distilled water (DW) was produced using a water distiller. Tap water (TW) and drain water (DRW) were collected at the Egypt-Japan University of Science and Technology (E-JUST) in Borg El-Arab City, Alexandria. Lake water (LW) was specifically sourced from Lake Mariout, while sea water (SW) was obtained from the Mediterranean Coast in Alexandria, Egypt. Furthermore, spinach leaves were collected from food residuals in a residential area in Alexandria, Egypt.

Methylene blue (C_16_H_18_ClN_3_S, ACS reagent grade, 90%), benzoquinone (C_6_H_4_O_2_, 97%), sodium azide (NaN_3_, 98%), chlorpyrifos (C_9_H_11_C_l3_NO_3_PS, 97%), and bromothymol blue dye (C_27_H_28_Br_2_O_5_S, ACS reagent grade, 95%) were supplied from Sigma-Aldrich. Ethanol (C_2_H_6_O, 99.9%, HPLC grade), hydrogen peroxide (H_2_O_2_, 30%), sodium hydroxide (ACS grade), paracetamol (C_8_H_9_NO_2_, 98%), and isopropanol (C_3_H_8_O, 99.8%, HPLC grade) were bought from Fisher Chemicals. Potassium peroxymonosulfate (KHSO_5_, 99.9%), hydrochloric acid (37%, analytical grade), and potassium persulfate (K_2_S_2_O_8_, 99.5%) were purchased from Merck. No pretreatment or purification steps were applied to any of the compounds or reagents used in the study. Deionized water, with a conductivity of 0.055 μS/cm, was prepared using the xCAD Plus Ultrapure Water Purification System (Thermo Scientific Co., USA) and utilized for the preparation of chemical solutions.

### Biochar synthesis and characterization

To synthesize BC from spinach leaves, a series of preparation steps were executed. Initially, the leaves were washed with deionized water to eliminate any undesired attached materials. Subsequently, they were subjected to drying in a furnace at 100 °C to eliminate excess moisture, which could otherwise interfere with the pyrolysis process and impede the efficient conversion of biomass into biochar (Li et al. 2023b). Following drying, the leaves were cut and ground into smaller fragments, thereby increasing their surface area. This enhancement facilitates improved contact between the biomass and the heat source during pyrolysis, promoting more efficient and uniform heat transfer (Seow et al. 2022). The resultant powder underwent sieving to eliminate larger particles that may be present after grinding, ensuring the uniform particle size of the produced biochar (Li et al. 2019). Subsequently, the sieved powder underwent pyrolysis at 500 °C in a nitrogen-purged muffle furnace for 2 h to obtain the BC. The absence of oxygen during this process prevents the biomass from undergoing complete combustion or burning (Armah et al. 2022). To eliminate contaminants from the produced BC particles, an extensive washing process was employed. Initially, the particles were treated with hydrochloric acid, followed by multiple water rinses. Ultimately, the BC particles were oven-dried at 70 °C for 24 h to complete the preparation process (El-Bestawy et al. 2023). A comprehensive depiction of the BC preparation steps is presented in Fig. (S1).

Chemical components of the BC were identified using X-ray fluorescence (XRF) analyzers and energy-dispersive X-ray spectroscopy (EDS). Crystallinity and phase identification were assessed through X-ray diffraction (XRD) analysis. Fourier-transform infrared spectroscopy (FTIR) was employed to identify chemical bonds in the synthesized BC. The morphology of the produced BC was monitored using transmission electron microscopy (TEM), high-resolution transmission electron microscopy (HRTEM), and selected area electron diffraction (SAED) images. Adsorption–desorption isotherm and pore size distribution data were acquired using a specific surface area analyzer known as the Brunauer, Emmett, and Teller (BET) instrument. The specific models of the characterization equipment are outlined in Table (S1).

### Experimental protocols and process optimization

The persulfate activation was conducted in a 250 mL Pyrex beaker at room temperature. A magnetic stirrer set at 950 rpm was employed to thoroughly mix the solution for a duration of 2 h, ensuring effective blending. A 400-W metal halide lamp, emitting light with a peak wavelength of 510 nm, was positioned 15 cm above the beaker to provide visible light to the system. The lamp remained fixed in this position throughout the experiment (Samy et al. 2023b).

The desired amounts of catalyst and oxidant doses were added to the beaker, along with 100 mL of the pollutant solution at a specified concentration. The initial pH of the resulting solution was adjusted using 0.1 M sodium hydroxide and/or 0.1 M hydrochloric acid as necessary. To establish adsorption–desorption equilibrium, the activators were mixed with the pollutant solution and allowed to stand in the dark for 30–60 min before the addition of the oxidant and/or illumination of the solution with light (Samy et al. 2019).

Suspension samples were extracted at various predetermined time intervals: 0, 20, 40, 60, 80, 100, and 120 min. For each extraction, 3 mL of the suspension was withdrawn using a syringe and subsequently centrifuged at 4000 rpm for 15 min. Following centrifugation, each sample underwent filtration through a 0.22 μm nylon syringe filter to prepare it for subsequent analysis.

In the control experiments, multiple experimental trials were conducted, employing different combinations of BC, visible light, and oxidants (PS, PMS, and HP) under specified conditions. The objective was to document the resulting removal efficiencies (REs%) for MB dye. The oxidant that exhibited the strongest activation and the system that achieved the highest RE% for MB dye were identified and subsequently selected for further experimentation.

To investigate the impact of pH, a series of experiments were undertaken with pH values set at 3, 5, 7, 9, and 11. All other conditions were maintained consistent with the parameters used in the preceding control experiments. Moreover, a comprehensive set of 15 experiments was carried out using the response surface methodology (RSM) and central composite design (CCD) to ascertain the optimal parameters for subsequent experiments.

To evaluate the enduring stability and potential for reuse of the synthesized BC, BC powder was collected and underwent a 24-h drying period in an oven after each 120-min cycle. This process was repeated for a total of five consecutive iterations. Throughout the five runs, the stability of BC was assessed by investigating the leaching of the main elements from the BC, as identified through XRF and EDS analyses. Additionally, the stability of the prepared BC was confirmed by comparing the results of FTIR and XRD for BC before treatment and after the 5th run. To evaluate the catalyst’s reusability, the breakdown efficiency of MB, the extent of total organic carbon (TOC) conversion, and the concentration of PS were examined over the course of the five runs.

Furthermore, the degradation of MB was investigated over a 2-h period in 100 mL samples of various water matrices, encompassing distilled water, tap water, lake water, drain water, and seawater. Additionally, the decontamination ratios of other pollutants, namely, bromothymol blue dye (BTB), paracetamol (PAC), and chlorpyrifos (CP), were assessed over a 120-min duration in 100 mL solutions containing each respective pollutant.

To identify the primary reactive oxygen species (ROS), scavenging experiments were carried out using different scavengers, including isopropanol (ISO), benzoquinone (BQ), ethyl alcohol (EtOH), and sodium azide (SA) (Xu et al. 2022). Furthermore, an investigation into the degradation of industrial effluent collected from a nearby textile plant was conducted.

The standardized procedures outlined in the “Standard Methods for the Examination of Water and Wastewater,” 23rd edition (Laura Bridgewater APHAAWWAWEF 2017), and the “Hach Water Analysis Handbook,” 3rd edition (Nollet and De Gelder 2013), were adhered to comply with regulations governing sample collection, preservation, storage, and analysis. A succinct summary of the testing equipment and methodologies employed for the assessment of untreated and treated water characteristics is provided in Table (S2). Additionally, Table (S3) presents the corresponding values for the characteristics of the raw water samples. The concentration (*C*) at time (*t*) was calculated using Eq. (1), while the RE% was determined at various time intervals using Eq. (2), where *A* represents the absorbance at time (*t*). On the other hand, *A*_o_ and *C*_o_ refer to the absorbance and concentration at the beginning of the experiment, respectively.

1 $$C=\left(A/{A}_{{\text{o}}}\right)*{C}_{{\text{o}}}$$

2 $${\text{RE}}\%=\left[\left({C}_{{\text{o}}}-C\right)/{C}_{{\text{o}}}\right]*100$$

The primary objective of the experimental design was to determine the optimal levels for the initial pollution concentration, catalyst dosage, and PS starting level, aiming to achieve the most effective removal of pollutants. RSM and CCD methods were employed to maximize the removal of MB as the dependent parameter. The optimum values for initial pollutant concentration, catalyst dosage, and PS starting level, serving as independent parameters, were identified through a total of 15 experimental runs, each lasting for 2 h.

The values and ranges of the operating parameters are detailed in Table 1. The impact of the independent parameters on the dependent variable exhibited a quadratic correlational pattern. This relationship between the dependent variable and the independent parameters can be mathematically represented by a quadratic equation (Samy et al. 2020b). The regression analysis was performed using Minitab@21 software, followed by an analysis of variance (ANOVA) to evaluate how well the model captured the variation in the data (Samy et al. 2020a).

**Table 1:** Fluctuations in values and ranges of operating parameters

Operating parameters	Units	Levels				
		− 2	− 1	0	1	2
MB concentration	mg/L	5	15	25	35	45
PS concentration	mM	0.1	0.15	0.2	0.25	0.3
Catalyst dose	g/L	0.05	0.075	0.1	0.125	0.15

## Results and discussion

### Characterization of synthetic biochar

**Fig. 1:**
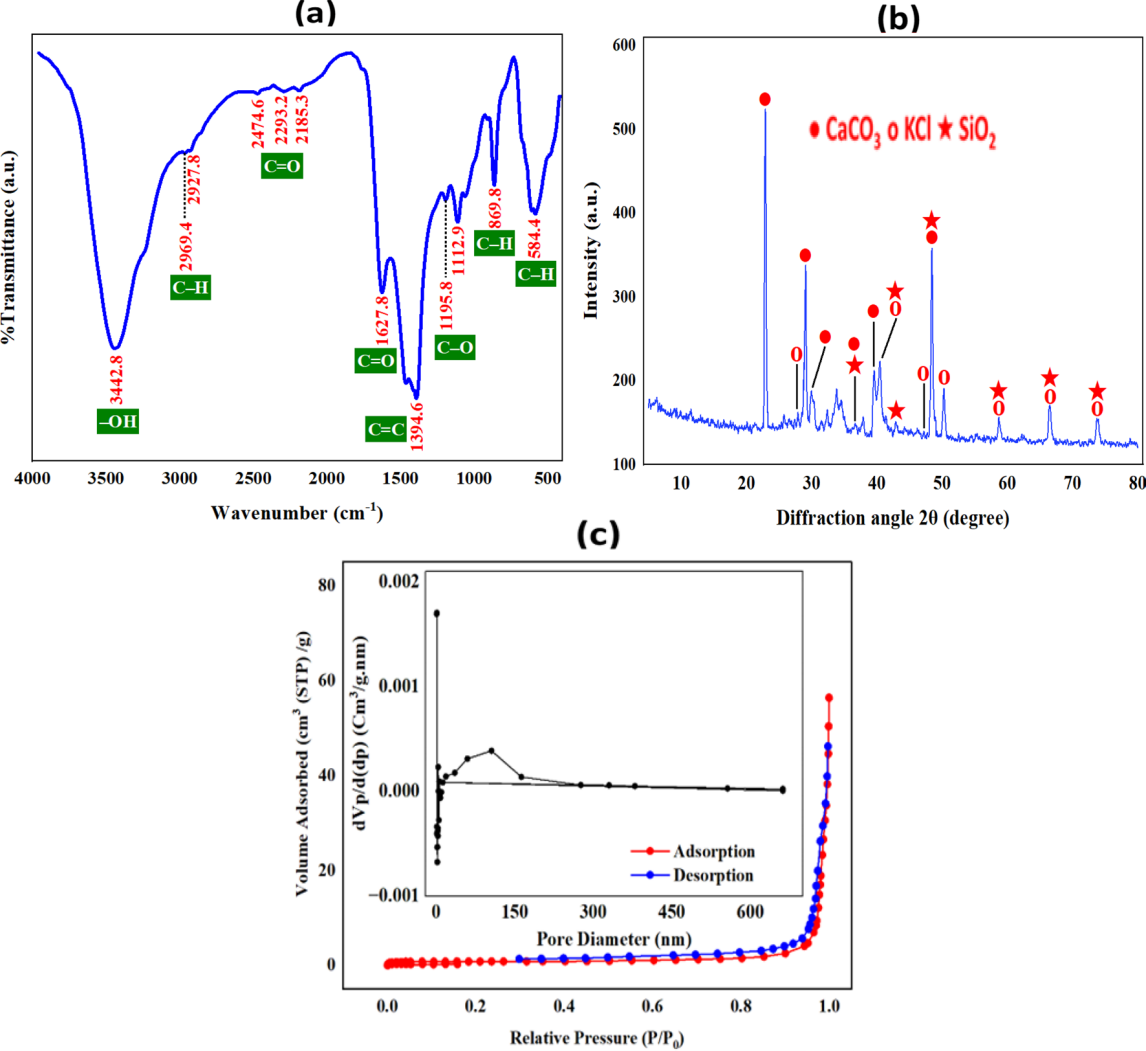
**a** FTIR spectra, **b** XRD pattern, and **c** adsorption–desorption isotherm and pore size distribution (insets) of the synthesized biochar

Figure 1a illustrates the FTIR spectra of the synthesized BC, revealing multiple absorption bands indicative of diverse functional groups containing oxygen, hydrogen, and carbon present on the BC surface. These functional groups are implicated in the effective activation of PS, as demonstrated by degradation experiments. The FTIR bands observed at 869.8 and 584.4 cm^−1^ can be attributed to the bending vibration of the C–H group (El-Bestawy et al. 2023). Bands at 1195.8 and 1112.9 cm^−1^ are associated with the C–O group (Mensah et al. 2022). The band at 1394.6 cm^−1^ corresponds to the C = C group (El-Bestawy et al. 2023). Bands at 1627.8, 2474.6, 2293.2, and 2185.3 cm^−1^ are attributed to the C = O group, following the findings of Li et al. (2014). Lastly, the broad band at 3442.8 cm^−1^ corresponds to the –OH group originating from water molecules adsorbed on the BC surface (Samy et al. 2023a). A comparative analysis with results from other studies in the literature (Table S4) validates that the major FTIR peaks observed in this study align with those previously reported.

The XRD pattern of the prepared BC sample (Fig. 1b) exhibited discernible peaks identified as potassium chloride (KCl), calcium carbonate (CaCO_3_), and silicon oxide (SiO_2_), determined through comparisons with standard XRD data from JCPDS #001–0837, ICSD #01–075-0296, and ICSD #01–076-094, respectively. This finding aligns with prior investigations conducted by Xu et al. (2022). The presence of CaCO_3_ and KCl in the prepared BC is plausibly linked to their presence in the soil used for cultivating spinach. Calcium fertilizers are commonly employed to regulate soil acidity, enhance soil physical and chemical properties, and supply calcium as a nutrient (Dzida and Jarosz 2010). Potassium chloride fertilizers, on the other hand, are utilized to efficiently provide potassium as a nutrient to crops (Borowski and Michalek 2009). Furthermore, silicon is typically found in the form of silicon dioxide (SiO_2_) rather than in its pure state, and it naturally occurs in leafy green vegetables like spinach. According to research by Powell et al. (2005), a 2-tablespoon serving of spinach contains approximately 4.1 mg of silicon dioxide (Powell et al. 2005). Standard XRD cards for KCl, CaCO_3_, and SiO_2_ are presented in Fig. (S2), while Table (S5) displays the 2θ° peak values obtained from XRD analysis of the BC sample, along with their corresponding Miller indices (hkl) sourced from reference cards. The absence of carbon peaks in the XRD pattern of the produced BC, as well as the amorphous structure, can be attributed to the interference of carbon peaks with other peaks and the overall amorphous nature of the material, as previously discussed by Kumi et al. (2020a).

The outcomes of the BET analysis applied to the synthesized BC revealed a specific surface area (*S*_BET_) of 2.8343 m^2^/g and a total pore volume of 0.0469 cm^3^/g. The relatively diminished *S*_BET_ is postulated to stem from the presence of tar-like compounds impeding the flow of substances within the pores, as proposed by Kumi et al. (2020b). Despite the limited surface area of BC, it is noteworthy that the removal of MB dye predominantly ensued through degradation by reactive species rather than adsorption. The nitrogen adsorption/desorption isotherm for the prepared BC, depicted in Fig. 1c, conforms to a characteristic type III isotherm according to the guidelines established by the International Union of Pure and Applied Chemistry (IUPAC) (Kumi et al. 2020b). The inset of Fig. 1c presents the pore size curve, revealing an average pore diameter of 105.54 nm in the prepared BC. This value exceeds the threshold of 50 nm, indicating that the predominant type of pores contributing to the total pore volume in the BC is macropores, in accordance with IUPAC guidelines.

**Fig. 2:**
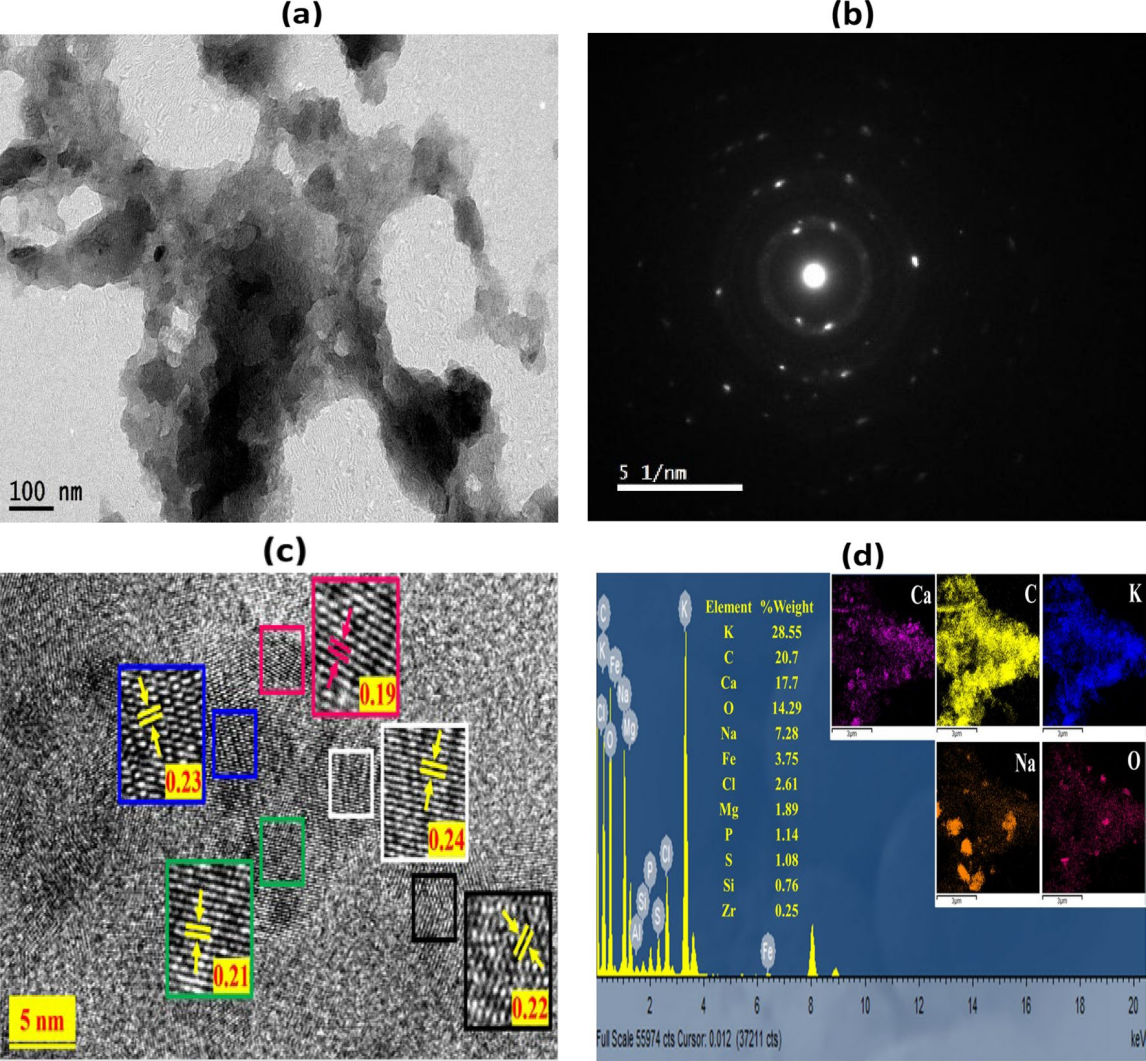
**a** TEM image, **b** SAED image, **c** HRTEM image, and **d** EDS pattern and elemental mapping (insets) of the synthesized biochar

The TEM image in Fig. 2a depicts the ovoidal-like morphology and porous structure of the BC particles. These visual observations align with the findings reported by El-Bestawy et al. (2023). The crystalline nature of the synthesized BC was substantiated through the SAED scan, as illustrated in Fig. 2b. Additionally, Fig. 2c, presenting the HRTEM image, provides supplementary evidence of the porous structure of BC. The EDS pattern and elemental mapping for BC are presented in Fig. 2d. The analysis reveals that BC is composed of various elements, including potassium, carbon, calcium, oxygen, sodium, iron, chloride, magnesium, phosphorus, sulfur, silicon, and zirconium, with weight percentages of 28.55, 20.7, 17.7, 14.29, 7.28, 3.75, 2.61, 1.89, 1.14, 1.08, 0.76, and 0.25%, respectively. The notable carbon content, constituting over 20% of the material by weight, validates the carbon-rich composition of BC. The elemental and oxide composition of the synthesized BC, as determined by XRF and reported in weight percent in Table (S6), is consistent with the composition analysis derived from XRD and EDS.

### Promoting efficient methylene blue dye decomposition

#### Comparative assessment of oxidants and activation techniques

**Fig. 3:**
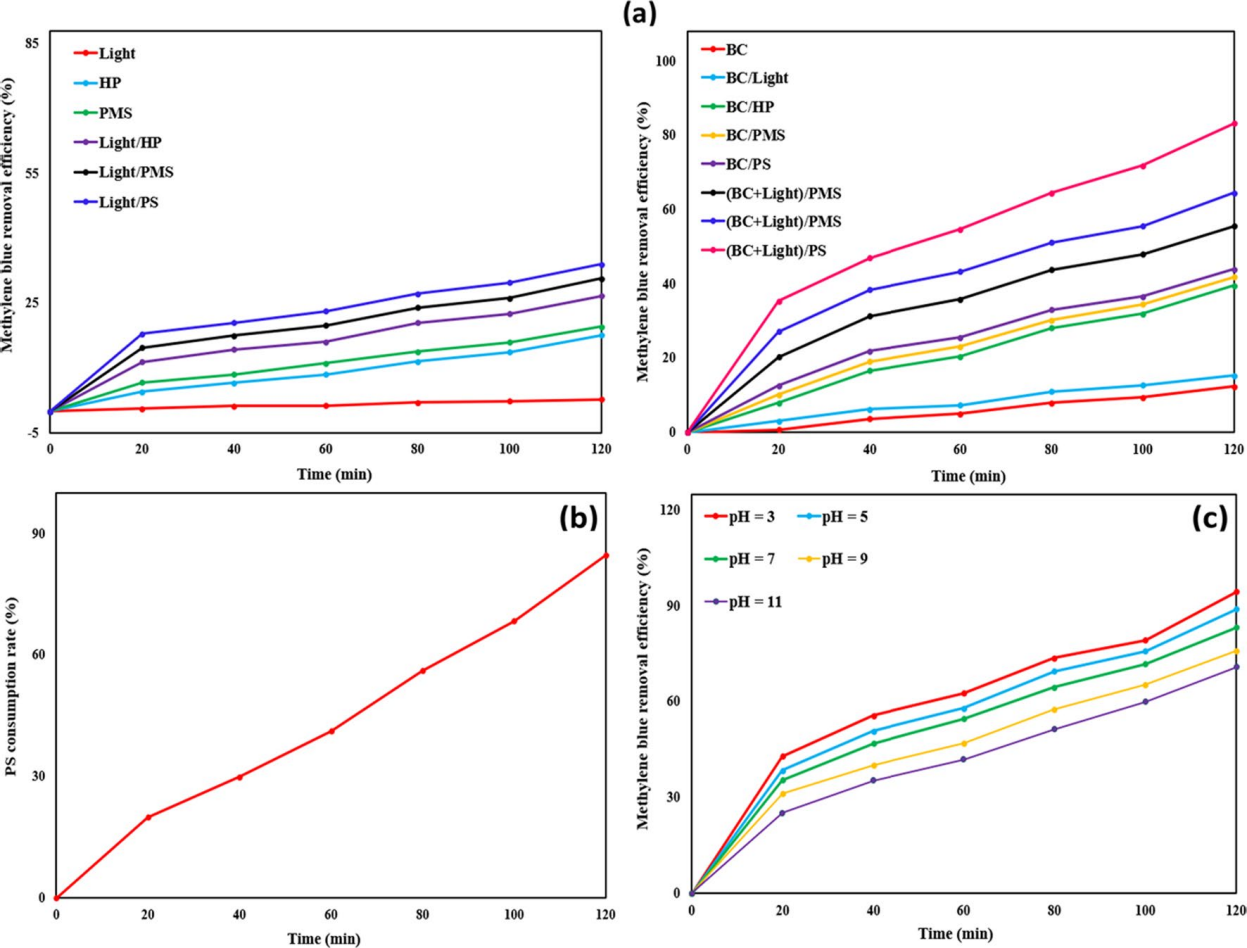
**a** Comparative analysis of MB dye RE% among different systems. **b** Consumption rates of PS. **c** Influence of pH on MB dye RE% over a 120-min/run using the BC@(PS + light) system (conditions: initial MB concentration = 25 mg/L, BC dosage = 0.1 g/L, initial PS concentration = 0.2 Mm, and run time = 120 min)

A series of control experiments, spanning a duration of 120 min, were conducted to assess the efficiency of the prepared BC and visible light in activating different oxidants, including PS, PMS, and HP, for the degradation of MB. The experimental conditions were standardized with an initial MB concentration of 25 mg/L, a catalyst dosage of 0.1 g/L, an initial oxidant concentration of 0.2 mM, and a pH value of 7. The experimental trials involved various combinations of BC, visible light, and different oxidants, with the corresponding MB dye REs% recorded and presented in Fig. 3a.

When utilizing BC alone, the recorded MB dye RE% was found to be 12.32%. This modest efficiency can be attributed to MB’s ability to either be adsorbed within the pores of BC or adhere to its surface, thereby impeding BC’s capacity to eliminate the contamination, as indicated by Samy et al. (2023b). Additionally, adsorption processes may occur through interactions such as π-π stacking between the O–H group in the synthesized BC and the aromatic group of MB or through hydrogen bonding involving the hydrogen in the hydroxyl group on the BC’s surface and the benzene ring in MB (Samy et al. 2022). The relatively low adsorption efficiency is likely linked to the limited surface area of the prepared BC (2.8343 m^2^/g). This constrained surface area resulted from the obstruction of pores by tar-like substances. Nonetheless, the diminished surface area ensured that the removal of MB predominantly occurred through degradation induced by reactive specie (Chowdhury et al. 2016).

The MB dye RE% was observed to be only 17.64, 19.5, and 23.16% when HP, PMS, and PS were individually employed without an activator. These outcomes suggest a relatively low effectiveness, which can be attributed to the lower oxidation potential of the oxidants compared to the radicals generated when the activator is engaged, underscoring the significance of activators in enhancing MB removal (Samy et al. 2020c). Upon incorporating light in conjunction with HP, PMS, and PS, higher MB dye RE% values were achieved compared to the use of oxidants alone, with recorded percentages of 26.64, 30.68, and 34.04, respectively. In contrast, the system employing light alone exhibited a substantially lower RE% of 2.73%. This finding further underscores the pivotal role of light in augmenting MB degradation by activating oxidants and generating radicals (Seyyedbagheri et al. 2023).

The activation of oxidants through the functional groups of the synthesized BC utilizing the BC/PS, BC/PMS, and BC/HP systems resulted in an improved degradation of MB, achieving levels of 44, 41.88, and 39.56%, respectively. This heightened efficiency can be ascribed to the production of sulfate and hydroxyl radicals (Xu et al. 2022). The combination of BC and light as oxidant activators in the (BC + light)/PMS, (BC + light)/HP, and (BC + light)/PS systems exhibited the highest efficiencies in MB degradation, reaching 64.52, 55.56, and 83.36, respectively. This superior performance can be attributed to the increased production of radicals resulting from the combined use of BC and light as activators, as elucidated by Zhang et al. (2022a).

Upon comparing all utilized oxidants, whether employed individually or in combination with light and/or BC, the MB degradation efficiency was found to be the lowest when utilizing HP. This can be attributed to the fact that the primary reactive species generated from HP are hydroxyl radicals, which have a shorter lifespan compared to the sulfate radicals produced when PS or PMS are employed (Luo et al. 2021).

Despite PMS demonstrating a higher MB dye RE% than HP, its effectiveness remained inferior to that of PS. The performance distinction between PS and PMS, despite both generating the same radical species, can be attributed to multiple factors. Firstly, PS possesses a higher redox potential (2.01 V) compared to PMS (1.82 V), as reported by Xiao et al. (2020). Furthermore, PS exhibits a longer O–O bond distance (1.497 Å) than that of PMS (1.453 Å), which facilitates the activation PS ions and enhances radical generation (Liu et al. 2023). Wang et al. (2020) and Hu et al. (2019) independently investigated the degradation of imidacloprid and tetracycline, respectively. Both studies reported that the PS/UV system exhibited a higher radical generation rate compared to the PMS/UV system (Wang and Wang 2018; Hu et al. 2019). In our study, experiments were conducted at pH 7, explaining why MB degradation was higher in the case of PS compared to PMS. This is because PS can generate radicals over a broader pH range, including both acidic and alkaline conditions, while PMS requires acidic pH conditions (below 6) for effective radical generation (Wang et al. 2020). The wider pH adaptability of PS enhances its applicability in various environmental and industrial scenarios. El-Bestawy et al. (2023) similarly found that the removal of atrazine at pH 7 was higher when utilizing the BC/PS system compared to the BC/PMS system (63% vs. 49%) (El-Bestawy et al. 2023).

The degradation efficiency of MB using BC alone, BC/light, light/PS, and (BC + light)/PS, was 12.32, 15.36, 34.04, and 83.36%, respectively (Fig. 3a). These findings confirm that the degradation of MB primarily occurs due to the activation of PS by light and the functional groups on the surface of BC. However, the limited improvement in MB removal efficiency observed when utilizing the BC/light system compared to BC alone (adsorption) can be attributed to the limited presence of metal oxides, as indicated in the XRF results in Table (S6), in the BC that can generate radicals after illumination by light. This confirms that the removal of MB was mainly due to adsorption and not photocatalysis in the case of using BC with light.

The influence of light on MB degradation in our research was investigated through control experiments utilizing various combinations of BC, light, and PS. Other studies, such as the one conducted by Gogoi et al. (2022a), confirmed the effect of light through experiments involving light on/off and intensity-dependent analyses (Gogoi et al. 2022a).

The (BC + light)/PS system, exhibiting the highest MB dye RE%, demonstrated significant activation of PS, with 84.7% consumption rate over a 2-h period (Fig. 3b). This observation provides additional evidence supporting the hypothesis that PS was activated, leading to the generation of reactive radicals through its interaction with PS-activating agents (BC and light), playing a critical role in the degradation mechanism. Based on the outcomes of control experiments, it has been determined that the (BC + light)/PS system exhibits the highest efficiency for degrading MB (83.36%). Therefore, this system will be employed for subsequent experiments.

#### Adjusting operational conditions for maximum methylene blue removal

The (BC + light)/PS system was employed to degrade MB under varying pH conditions (Fig. 3c). With an initial MB concentration of 8.5 mg/L, a catalyst dose of 0.15 g/L, and an initial PS concentration of 0.3 mM, the experiment was conducted for 120 min. The results indicated that higher pH values had a suppressive effect on the degradation of MB, resulting in degradation percentages of 94.53, 89.1, 83.36, 75.97, and 70.9% at pH 3, 5, 7, 9, and 11, respectively. Similar trends were reported by Zhu et al. (2023) in the breakdown of arsenic using biochar made from sewage sludge (Zhu et al. 2023). Additionally, comparable findings were observed by Samy et al. (2023a), utilizing toner powder waste for MB degradation via PS activation (Samy et al. 2023b). Elevated pH values can impede the decomposition of PS in the solution, resulting in a diminished formation of sulfate and hydroxyl radicals (Samy et al. 2023a). Conversely, under acidic conditions, a greater abundance of sulfate radicals is generated, thereby increasing the production of hydroxyl radicals and fostering higher rates of MB degradation (Miserli et al. 2022). The minimal variation in degradation efficiency observed across the tested pH values indicates that the (BC + light)/PS system can achieve significant degradation performance consistently, irrespective of pH conditions. As a result, subsequent experiments were conducted at a neutral pH of 7.

The second-order model, as expressed in Eq. (3), establishes a mathematical relationship between the dependent parameter (MB dye RE%) and the independent operating conditions employed in the (BC + light)/PS system. The coefficient of determination (*R*^2^) obtained from this equation serves as a metric for assessing the adequacy of the model’s fit.

3 $$\mathrm{RE\% }= 61.7 + 0.105X+ 142Y+ 161Z- 0.00773{X}^{2}- 102{Y}^{2}- 304{Z}^{2}- 1.6XY- 1.17XZ- 163 YZ$$

where *X* represents the initial concentration of MB (mg/L), *Y* denotes the initial dosage of PS (mM), and *Z* represents the dose of catalyst (mg/L).

Table 2 presents the anticipated and observed efficiencies of MB removal when employing the (BC + light)/PS system under various conditions. Meanwhile, Table 3 furnishes evidence supporting the utility of the proposed model, as indicated by the results of the ANOVA analysis. *F* values of 93.6, 9.27, and 1.69 were obtained for MB initial concentration, PS initial concentration, and BC dose, respectively. These values suggest that MB concentration had the most substantial influence on degradation performance, followed by PS concentration, with BC dose exerting the weakest effect.

**Table 2:** Experimental and model-derived MB dye REs% under various operational conditions

Run	Parameters’ codes			Parameters’ real values			Methylene blue RE%	
	MB conc. (mg/L)	PS conc. (mM)	BC dose (g/L)	MB conc. (mg/L)	PS conc. (mM)	BC dose (g/L)	Measured	Predicted
1	1	1	1	35	0.25	0.125	75.09	76.19
2	1	− 1	1	35	0.15	0.125	71.37	73.71
3	1	1	− 1	35	0.25	0.075	73.60	75.27
4	1	− 1	− 1	35	0.15	0.075	69.86	71.97
5	− 1	1	1	15	0.25	0.125	94.40	92.75
6	− 1	− 1	1	15	0.15	0.125	88.27	87.07
7	− 1	1	− 1	15	0.25	0.075	92.53	90.65
8	− 1	− 1	− 1	15	0.15	0.075	84.80	84.16
9	− 2	0	0	5	0.2	0.1	91.20	93.97
10	2	0	0	45	0.2	0.1	68.73	65.23
11	0	0	2	25	0.2	0.15	84.08	83.85
12	0	0	− 2	25	0.2	0.05	80.52	80.01
13	0	2	0	25	0.3	0.1	85.76	86.16
14	0	− 2	0	25	0.1	0.1	78.32	77.18
15	0	0	0	25	0.2	0.1	83.36	82.69

**Table 3:** Assessment of the significance of the model

Source	DF	Sum of squares	Mean square	*F* value	*P* value
Model	9	938.741	104.305	11.81	0.007
Linear	3	923.681	307.894	34.86	0.001
*X* (mg/L)	1	826.836	826.836	93.60	0.000
*Y* (mM)	1	81.920	81.920	9.27	0.029
*Z* (g/L)	1	14.925	14.925	1.69	0.250
Square	3	8.912	2.971	0.34	0.801
*X* (mg/L)*X (mg/L)	1	6.620	6.620	0.75	0.426
*Y* (mM)*Y (mM)	1	0.719	0.719	0.08	0.787
*Z* (g/L)*Z (g/L)	1	0.399	0.399	0.05	0.840
2-way interaction	3	6.147	2.049	0.23	0.871
*X* (mg/L)*Y (mM)	1	5.135	5.135	0.58	0.480
*X* (mg/L)*Z (g/L)	1	0.681	0.681	0.08	0.792
*Y* (mM)*Z (g/L)	1	0.332	0.332	0.04	0.854
Error	5	44.167	8.833		
Total	14	982.909			

**Fig. 4:**
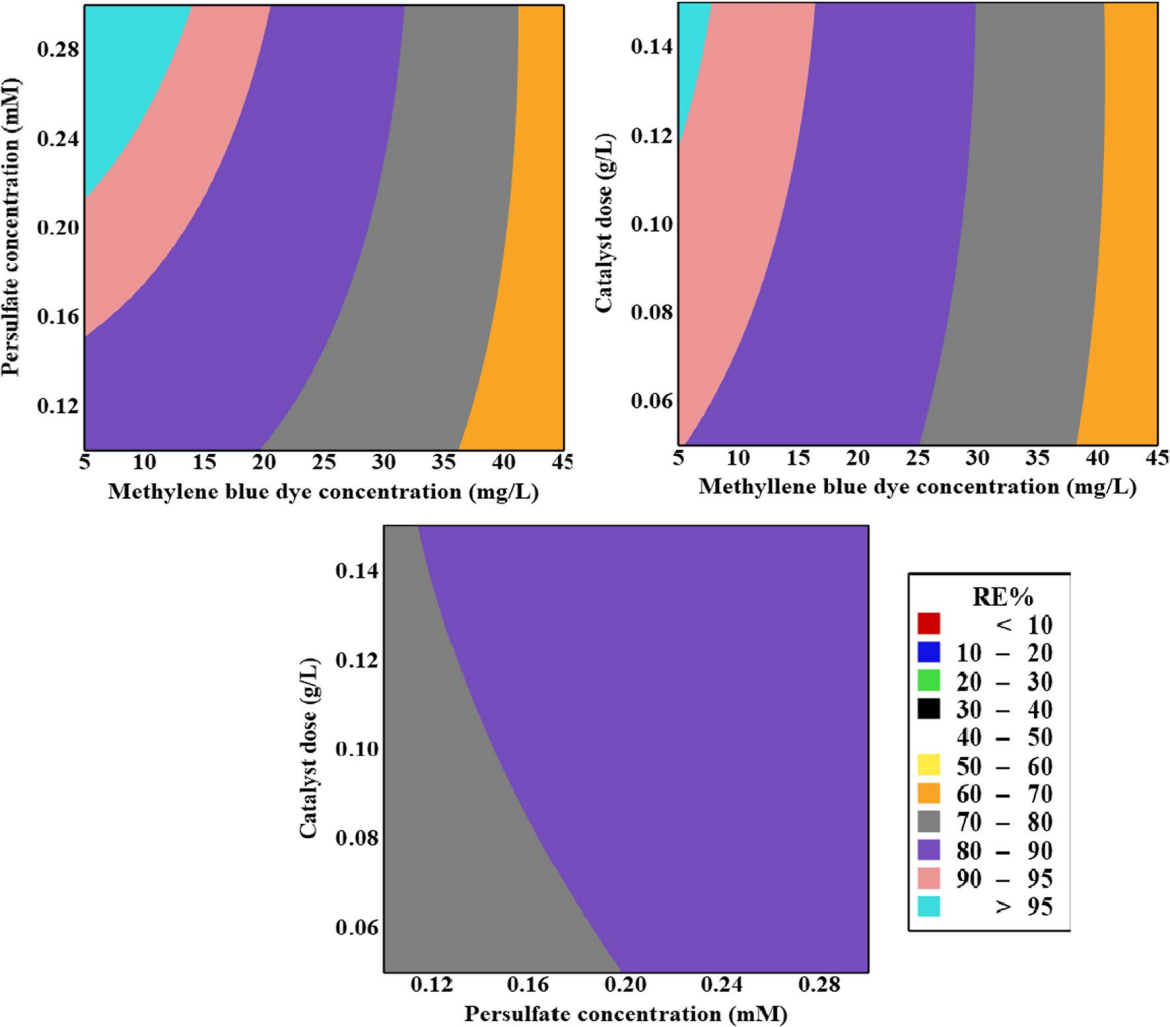
Contour plots showing the effect of operating conditions on MB dye RE%

The degradation rates of MB at various BC doses, PS concentrations, and MB concentration levels are illustrated in Fig. 4. The findings indicated that the MB degradation ratio attained its peak when the MB concentration was 8.5 mg/L or lower. At lower MB concentrations, a surplus of radicals was generated, enabling the effective decomposition of MB at a high rate. However, at higher MB concentrations, more radicals were required to degrade the increased amount of MB, which the system could not adequately provide (Samy et al. 2020c).

Given that free radicals govern the decomposition of MB, elevating the PS concentration enhanced degradation efficiency by supplying more sulfate and hydroxyl radicals (Saien and Jafari 2022). However, the results indicated that PS concentrations above 0.3 mM led to a reduction in the system’s efficiency. This is attributed to the inhibitory effect of excessively high PS concentrations, as illustrated by Eqs. (4) and (5). These equations depict how an excess of PS can impede radical generation rather than promoting it (Adel et al. 2020).

4 $${{SO}_{4}}^{\bullet -}+{S}_{2}{{O}_{8}}^{2-}\to {S}_{2}{{O}_{8}}^{\bullet -}+{{SO}_{4}}^{2-}$$

5 $${HO}^{\bullet }+{S}_{2}{{O}_{8}}^{2-}\to {S}_{2}{{O}_{8}}^{\bullet -}+{OH}^{-}+{{O}_{2}}^{\bullet -}$$

The escalation of the BC dose up to 0.15 g/L, identified as the optimal BC dose, resulted in an upward trajectory in the degradation of MB. This phenomenon was attributed to the abundance of active sites capable of activating PS (El-Bestawy et al. 2023). However, the subsequent decrease in active surface area, induced by particle agglomeration, offset the positive impact of overdosing. This mitigating effect prevented further enhancement of degrading performance by quenching the generated radicals (Ahmed et al. 2021).

As the catalyst dose increased, its efficiency in facilitating light-driven activation of both the catalyst surface and PS anions diminished at a proportional rate (Shokri 2018). Table 4 delineates the optimized operational parameters of the model alongside their corresponding MB degradation efficiency. The results indicate a slight disparity between the observed removal effectiveness during experimentation and the predicted values, underscoring the practical applicability of the model.

**Table 4:** The optimum working parameters as well as the anticipated and actual MB removal rates

Parameters	Optimum values
MB conc. (mg/L)	8.5
PS conc. (mM)	0.3
Catalyst dose (g/L)	0.15
Expected MB RE% under the optimum values after 120 min	99.86%
Real MB RE% within 120 min using the above-stated conditions	99.02%

#### Efficiency evaluation of the (BC + light)/PS system on various pollutants

**Fig. 5:**
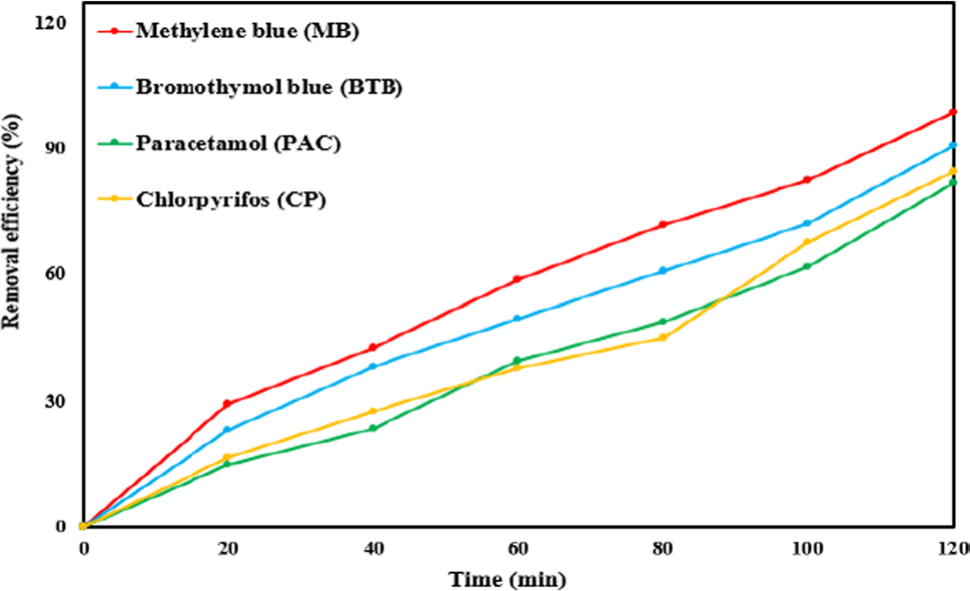
Degradation of MB, BTB, PAC, CP using the BC@(PS + light) system under the optimum conditions (initial pollutants’ concentrations = 8.5 mg/L, BC dosage = 0.15 g/L, initial PS concentration = 0.3 mM, and run duration = 120 min)

The investigation assessed the efficiency of the (BC + light)/PS system in degrading persistent contaminants, including insecticides (chlorpyrifos, CP), medications (paracetamol, PAC), and bromothymol blue dye (BTB). The methodologies and instruments utilized to measure the absorbance of CP, PAC, and BTB before and after treatment are documented in Table (S2). Concentration and removal efficiencies were computed using the formulas specified in Eqs. (1) and (2). The study was carried out under predetermined optimal conditions, encompassing an initial pollutant concentration of 8.5 mg/L, a catalyst dosage of 0.15 g/L, an initial PS concentration of 0.3 mM, and a pH level of 7. The experimental duration was 120 min. The degradation efficiencies of the pollutants BTB, PAC, and CP were observed to be lower than those of MB (99.02%), specifically, 90.82%, 81.88%, and 84.82%, respectively, as depicted in Fig. 5. The variations in degradation efficiencies among the pollutants can be attributed to the optimization process being exclusively conducted for MB and not for the other contaminants. For each pollutant, separate optimization procedures should be undertaken to determine the optimum catalyst dose, pH level, PS concentration, and initial pollutant concentration to maximize efficiency. Nevertheless, the (BC + light)/PS system demonstrated exceptional performance in activating PS and facilitating the degradation of various organic pollutants, as evidenced by the high degradation ratios achieved.

To validate the effectiveness of the proposed system in this study, a comparison was undertaken to assess the catalytic performance of the (BC + light)/PS system in conjunction with other systems incorporating biochars sourced from various origins. The evaluation specifically centered on their capacities to activate PS and degrade refractory dyes. The outcomes of this comparative analysis are detailed in Table (S7).

### Evaluating the stability and reusability of the prepared biochar

**Fig. 6:**
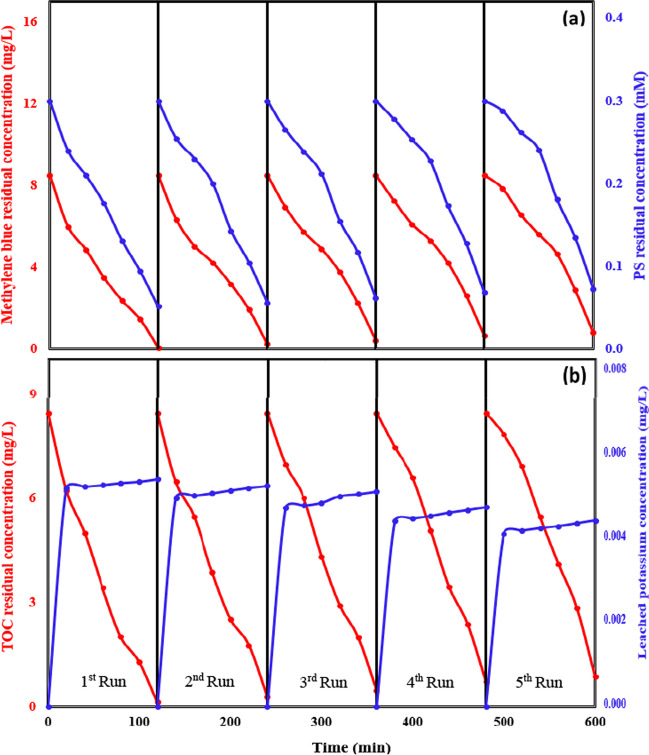
Evaluation of (**a)** MB dye and PS RCs and (**b)** TOC and leached potassium RCs, over five successive runs using the BC@(PS + light) system (conditions: initial MB concentration = 8.5 mg/L, BC dosage = 0.15 g/L, initial PS concentration = 0.3 mM, and run duration = 120 min)

To assess the stability and recyclability of the prepared BC, BC powder was collected and subjected to a 24-h drying process in an oven after each cycle. This approach enabled the catalyst powder to be utilized for five consecutive runs, with each run lasting 120 min, under the optimum conditions utilizing the (BC + light)/PS system. These conditions involved an initial concentration of 8.5 mg/L for MB, a catalyst dosage of 0.15 g/L, an initial PS concentration of 0.3 mM, and a pH value of 7. The degradation percentages of MB after each run were observed to be 99.02, 96.97, 94.94, 92, and 90.35%, respectively (Fig. 6a). Additionally, the recorded consumption rates of PS during the five repetitive cycles were observed to be 82.7, 81.33, 79.33, 77, and 75.67% (Fig. 6a). These findings serve as evidence for the activation of PS by the (BC + light)/PS system. Further, the corresponding TOC mineralization efficiencies of MB after each run were found to be 98.46, 96.57, 94.33, 91.37, and 89.72%, respectively (Fig. 6a). The consecutive runs exhibit a slight decrease in MB dye RE% and TOC mineralization efficiencies, indicating that the prepared BC can be reused. However, this decline in successive runs can be attributed to the blockage of active sites on the biochar particles. This blockage is caused by the agglomeration of MB and the formation of byproduct molecules in subsequent cycles (Niu et al. 2021). Additionally, there may be a loss of biochar particles during the sampling process, as noted by Tolba et al. (2019).

The stability of the prepared BC was confirmed through the analysis of its chemical composition, chemical structure, and functional groups using XRF, XRD, and FTIR techniques, respectively, after the 5th run, as depicted in Table (S6) and Fig. (S3), respectively. The results indicated that there were no noteworthy alterations, thereby confirming the stability of the prepared BC. Furthermore, during the five experimental runs, the leaching process of potassium (K) from BC to water was investigated, considering its prominence as the primary metal in BC, as evidenced by the XRF results (Table S6) and EDS elemental mapping results presented in Fig. 2d. The potassium absorbance analysis method used is stated in Table (S2). The results revealed the concentration of leached potassium ions after each run, which were as follows: 5.56, 5.4, 5.26, 4.88, and 4.55 µg/L, respectively (Fig. 6b). These findings confirm the considerable stability of the prepared BC, as demonstrated by the consistent leaching behavior of potassium throughout the five runs. The World Health Organization (WHO) states that adults should consume more than 3000 mg of potassium per day (WHO 2022). This information suggests that the leaching of potassium cannot be regarded as a secondary form of pollution.

To validate the importance of residual iron, the concentrations of leached iron were measured using ICP-MS. The results, depicted in Fig. (S4), showed that the leached iron concentrations were 0.22, 0.18, 0.15, 0.13, and 0.11 mg/L in the five repetitive cycles. The low concentrations of leached iron ions could contribute to the activation of PS which improved the degradation performance as described in Eqs. (11) and (12) (Samy et al. 2023a). Furthermore, the results showed a decrease in the leached iron concentration with cycles. Therefore, a slight reduction in the removal efficiency was observed in the repetitive cycle. The decrease in Fe^2+^ concentration could be attributed to its consumption during the PS activation process and conversion to Fe^3+^ that might precipitate as iron hydroxides. However, the limited improvement in MB dye RE% observed when utilizing the BC/light system compared to BC alone (adsorption), as depicted in Fig. 3a, can be attributed to the limited presence of metal oxides (e.g., iron oxide) in the BC that can generate radicals after illumination by light. This confirms that the removal of MB was mainly due to adsorption and not photocatalysis by the residual iron or other metal oxides in the case of using biochar with light. The study by Gogoi et al. (2022b) demonstrated the role of iron oxide in enhancing the photocatalytic activity of polypyrrole polymer under visible light (Gogoi et al. 2022b). Importantly, the leached iron concentration observed in our study does not pose a secondary pollution concern, as the dissolved concentrations fall within the allowable range (Fe < 0.3 mg/L) established by WHO (WHO 2022b).

### Assessment of methylene blue dye degradation in diverse water matrices

This experiment was conducted to evaluate the influence of various water matrices, specifically distilled water (DW), tap water (TW), sea water (SW), lake water (LW), and drain water (DRW), on the removal efficiency of MB dye. The degradation of MB can be adversely affected by the presence of inorganic ions (such as nitrates, sulfates, and chlorides) and dissolved organic matter (DOM) within the water matrices (Tran et al. 2019). Comprehensive information regarding the physicochemical characteristics of the diverse water matrices can be found in Table (S3).

The degradation of MB was examined over a duration of 120 min employing the (BC + light)/PS system under optimized conditions. These optimal conditions included an initial MB concentration of 8.5 mg/L, a catalyst dose of 0.15 g/L, a starting PS concentration of 0.3 mM, and a pH value of 7.

**Fig. 7:**
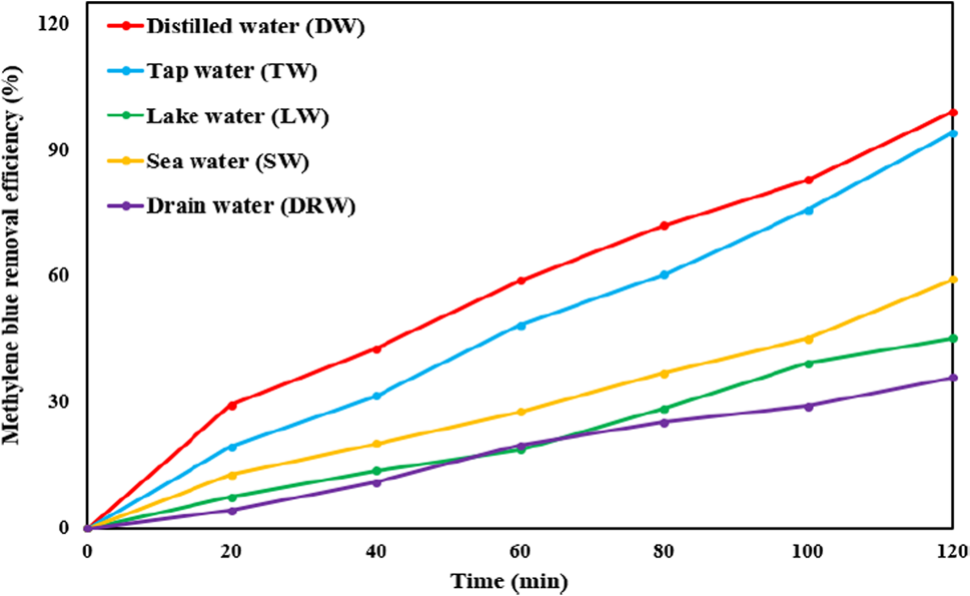
Assessment of the performance of the BC@(PS + light) system for MB dye removal in different water matrices (conditions: initial MB concentration = 8.5 mg/L, BC dosage = 0.15 g/L, initial PS concentration = 0.3 mM, and run duration = 120 min)

As depicted in Fig. 7, DW exhibited the highest degradation ratio of MB at 99.02%, whereas the ratios declined for other water matrices, ranging from TW at 94.12%, SW at 59.26%, and LW at 45.18%, with DRW demonstrating the lowest ratio at 35.85%. The reduced degradation ratios observed in certain SW, LW, and DRW matrices can be attributed to the varied concentrations of DOM and inorganic salts. These factors may have impeded the effectiveness of radicals, diminishing their capacity for the degradation of MB, as indicated by Samy et al. (2021).

### Methylene blue degradation mechanism

**Fig. 8:**
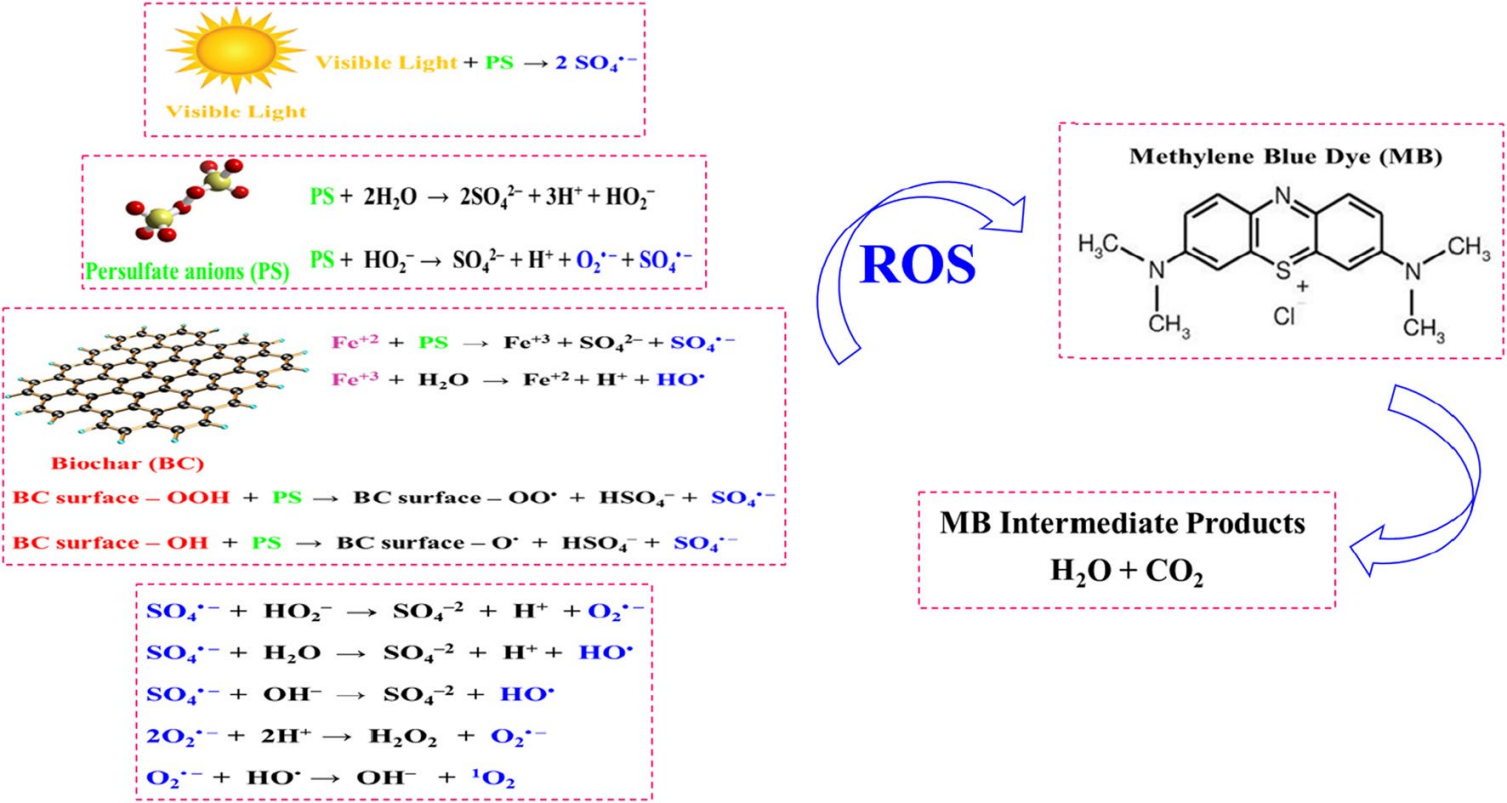
MB degradation mechanism utilizing the BC@(PS + light) system

The mechanism through which MB undergoes degradation utilizing the (BC + light)/PS system is elucidated in Fig. 8. Equations (6) and (7) delineate the degradation pathway of MB in the presence of PS alone, without light irradiation. In this process, PS reacts with water (H_2_O), forming hydroperoxyl ions (HO_2_^−^), which subsequently interact with PS to generate sulfate radicals (SO_4_^• −^) and superoxide radicals (O_2_^• −^) (Deng et al. 2022). As per Eq. (8), visible light alone initiates the activation of PS, leading to the production of (SO_4_^• −^) by breaking the peroxide bond (Su et al. 2019).

As depicted in Fig. 1a of the FTIR results, the surface of BC reveals the presence of oxygen-containing functional groups, including carbonyl and hydroxyl groups. These functional groups serve as electron donors, promoting the transfer of electrons from the surface of BC to PS. This electron transfer facilitates the activation of PS, resulting in the generation of (SO_4_^• −^), as outlined in Eqs. (9) and (10) (Pervez et al. 2019). Additionally, the free π electrons inherent in the sp2 hybrid structure of carbon materials have the potential to migrate from the sp2 orbitals of BC to PS, thereby activating PS and leading to the generation of additional (SO_4_^• −^), as demonstrated by Xu et al. (2020). Moreover, structural features such as pores, bending, and edge flaws within the BC’s composition may generate dangling sigma-bonds, expediting the electron transition from the BC surface to PS. This process activates PS and augments radical generation, as reported by Ouyang et al. (2019).

Moreover, the identification of iron in the EDS elemental mapping (Fig. 2d) confirms its presence, which may contribute to the activation of PS, potentially resulting in heightened generation of and (SO_4_^• −^) hydroxyl radicals (HO^•^), as illustrated in Eqs. (11) and (12). Furthermore, the metal oxides identified in the BC, as revealed by the XRF results in Table (S6), have the capability to be excited under visible light, leading to the generation of radicals and enhancing the degradation of MB. However, it is crucial to note that this contribution is comparatively limited when compared to the impact of PS activation by BC and light, as evidenced in the results of the control experiments.

In our study, the photobleaching of MB was not facilitated using an electron donor. Moreover, in the event of potential photobleaching, our system operates in an open configuration, where oxygen is available to oxidize the leuco form (colorless) back to MB. It is important to emphasize that the primary objective of introducing light into the degradation system is to activate PS (Eq. (8)), in addition to its activation by the functional groups attached to the BC (Eqs. (9) and (10)). This is distinct from illuminating BC itself, as BC is recognized as a weak semiconductor with limited efficiency in light absorption and the generation of sufficient reactive oxygen species through photocatalysis. This limitation stems from the relatively low weight ratios of metal oxides, including ZnO, CuO, and Fe_2_O_3_, which function as photocatalysts in the synthesized BC, as outlined in Table (S6). Despite this limitation, the radicals generated through the activation of PS exhibit a continuous capacity to oxidize MB molecules, converting them into innocuous compounds like water and carbon dioxide. The outcomes of the control experiments affirm that the degradation of MB predominantly arises from the activation of PS. The marginal enhancement in MB removal efficiency observed when employing the BC/light system, as opposed to BC alone (adsorption), can be ascribed to the restricted presence of metal oxides in the BC. These metal oxides have the capability to generate radicals upon exposure to light. This observation confirms that the predominant mechanism for MB removal is adsorption rather than photocatalysis when employing BC with light. Moreover, when subjected to light alone, MB exhibited only limited degradation without a loss of color. These findings substantiate that photobleaching of MB did not occur in the experimental system. In contrast, Lee et al. (2005) propose that photobleaching of colored organic species, such as MB, can occur under visible light illumination when a semiconductor and an electron donor, like triethanolamine, are present. Additionally, MB itself can absorb photons and transition to an excited state. However, photobleaching of MB does not occur in its pure form; an electron donor is essential to bleach MB and convert it to the leuco form. Conversely, MB can be regenerated from the leuco form through oxidation by oxygen (Lee et al. 2005).

Additionally, as delineated in Eqs. (13)–(15), hydroxyl ions (OH^−^), (H_2_O), and (HO_2_^−^) can combine with water to generate (HO^•^) and (O_2_^• −^) radicals (Deng et al. 2022). Moreover, (O_2_^• −^) radicals may interact with (H_2_O) to produce singlet oxygen (^1^O_2_), which plays a role in MB degradation through a non-radical pathway, as elucidated in Eqs. (16) and (17) (Li et al. 2022b). As expressed in Eq. (18), ROSs mineralize the MB dye into intermediates, (H_2_O), and carbon dioxide (CO_2_) (Zhang et al. 2021).

6 $${{\text{S}}}_{2}{{{\text{O}}}_{8}}^{2-}+{2{\text{H}}}_{2}{\text{O}}\to {{2{\text{SO}}}_{4}}^{2-}+{3{\text{H}}}^{+}+{{{\text{HO}}}_{2}}^{-}$$

7 $${{\text{S}}}_{2}{{{\text{O}}}_{8}}^{2-}+{{{\text{HO}}}_{2}}^{-}\to {{{\text{SO}}}_{4}}^{2-}+{{\text{H}}}^{+}+{{{\text{O}}}_{2}}^{\bullet -}+{{{\text{SO}}}_{4}}^{\bullet -}$$

8 $${{\text{S}}}_{2}{{{\text{O}}}_{8}}^{-2}+{\text{hv}}\to {{2\mathrm{ SO}}_{4}}^{\bullet -}$$

9 $$\mathrm{BC surface}-{\text{OOH}}+{{\text{S}}}_{2}{{{\text{O}}}_{8}}^{-2}\to \mathrm{BC surface}-{\text{OO}}\bullet +{{{\text{HSO}}}_{4}}^{-}+{{{\text{SO}}}_{4}}^{\bullet -}$$

10 $$\mathrm{BC surface}-{\text{OH}}+{{\text{S}}}_{2}{{{\text{O}}}_{8}}^{-2}\to \mathrm{BC surface}-{\text{O}}\bullet +{{{\text{HSO}}}_{4}}^{-}+{{{\text{SO}}}_{4}}^{\bullet -}$$

11 $${{\text{Fe}}}^{+2}+{{\text{S}}}_{2}{{{\text{O}}}_{8}}^{2-}\to {{\text{Fe}}}^{+3}+{{{\text{SO}}}_{4}}^{2-}+{{{\text{SO}}}_{4}}^{\bullet -}$$

12 $${{\text{Fe}}}^{+3}+{{\text{H}}}_{2}{\text{O}}\to {{\text{Fe}}}^{+2}+{{\text{H}}}^{+}+{{\text{HO}}}^{\bullet }$$

13 $${{{\text{SO}}}_{4}}^{\bullet -}+{{{\text{HO}}}_{2}}^{-}\to {{{\text{SO}}}_{4}}^{-2}+{{\text{H}}}^{+}+{{{\text{O}}}_{2}}^{\bullet -}$$

14 $${{{\text{SO}}}_{4}}^{\bullet -}+{{\text{H}}}_{2}{\text{O}}\to {{{\text{SO}}}_{4}}^{-2}+{{\text{H}}}^{+}+{{\text{HO}}}^{\bullet }$$

15 $${{{\text{SO}}}_{4}}^{\bullet -}+{{\text{OH}}}^{-}\to {{{\text{SO}}}_{4}}^{-2}+{{\text{HO}}}^{\bullet }$$

16 $${{2{\text{O}}}_{2}}^{\bullet -}+{2{\text{H}}}^{+}\bullet {{\text{H}}}_{2}{{\text{O}}}_{2}+{{{\text{O}}}_{2}}^{\bullet -}$$

17 $${{{\text{O}}}_{2}}^{\bullet -}+{{\text{HO}}}^{\bullet }\to {{\text{OH}}}^{-}+{}^{1}{{\text{O}}}_{2}$$

18 $${{\text{ROS}}}_{{\text{s}}}+{\text{MB}}\to \mathrm{Intermediate products}+{{\text{CO}}}_{2}+{{\text{H}}}_{2}{\text{O}}$$

**Fig. 9:**
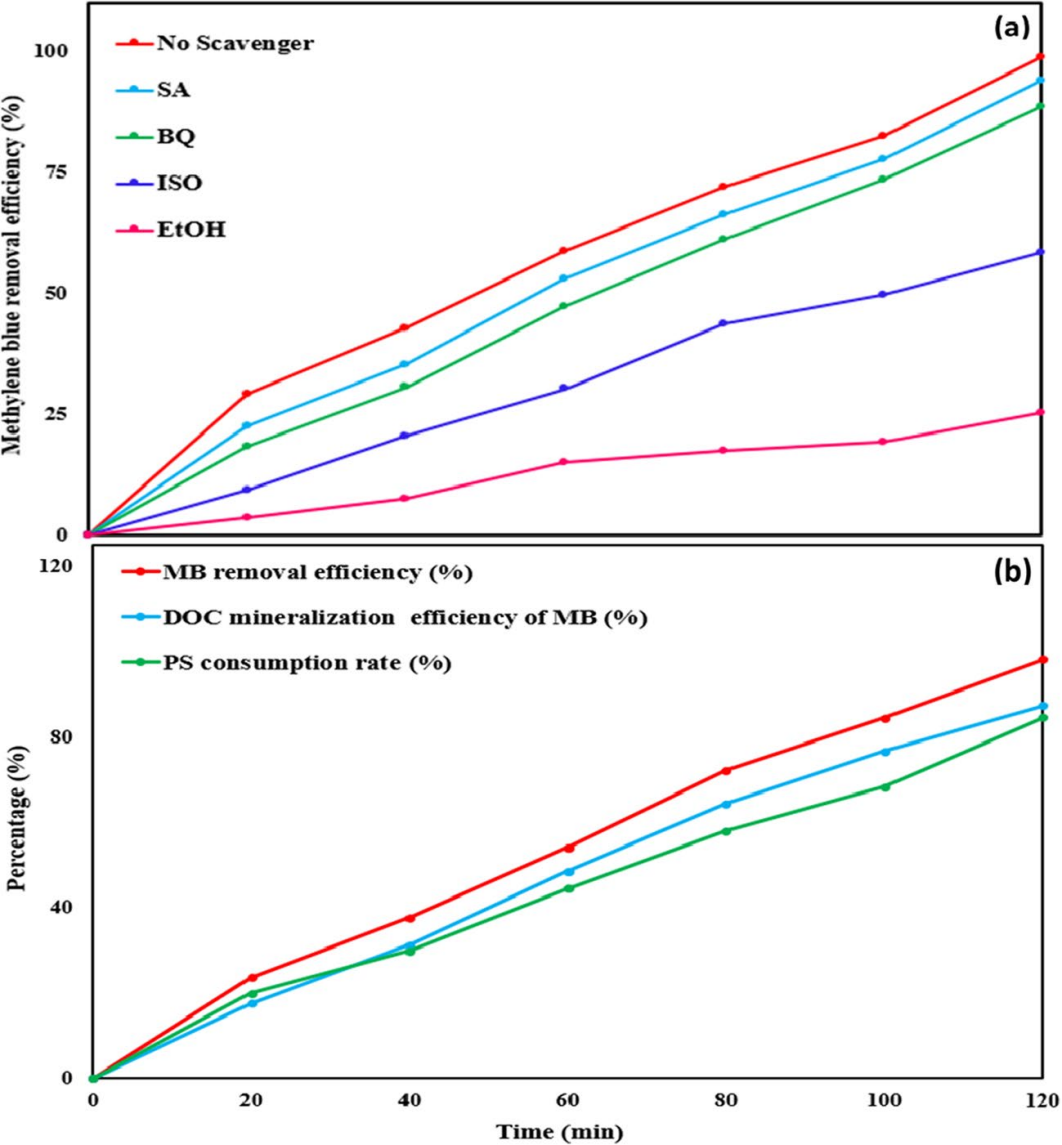
**a** Impact of different quenchers on MB removal using the BC@(PS + light) system. **b** Assessment of MB and DOC REs% and PS consumption rate in real textile wastewater utilizing the BC@(PS + light) system at optimum conditions

To ascertain the predominant reactive species, a range of scavengers was introduced. These quenchers, namely, ethanol (EtOH), isopropanol (ISO), benzoquinone (BQ), and sodium azide (SA), were deliberately selected to counteract sulfate, hydroxyl, superoxide radicals, and non-radical singlet oxygen, respectively (El-Bestawy et al. 2023). Figure 9a illustrates the influence of various scavengers on the degradation rate of MB employing the (BC + light)/PS system. The experiment was conducted over a period of 120 min, with an initial MB concentration of 8.5 mg/L, a catalyst dose of 0.15 g/L, a starting PS concentration of 0.3 mM, a pH value of 7, and a scavenger concentration of 300 mM. In the presence of SA, BQ, ISO, and EtOH, the degradation efficiencies of MB exhibited reductions to 94.12%, 88.71%, 58.47%, and 25.41%, respectively, compared to the scenario without any quencher present (99.02%). The substantial decrease in the removal efficiency of MB upon the introduction of EtOH and ISO confirms the predominant role of sulfate and hydroxyl radicals compared to other radicals. The decreased rate of MB decomposition observed with the use of SA provides evidence for the negligible contribution of the non-radical pathway in the process. The addition of BQ resulted in a minor decrease in MB removal, indicating the involvement of superoxide radicals in the degradation process.

### Treatment of real textile wastewater to remove methylene blue dye

The assessment of the (BC + light)/PS system’s performance and efficiency in treating textile industrial wastewater was conducted under optimal conditions. These conditions encompassed an initial MB concentration of 8.5 mg/L, a catalyst dose of 0.15 g/L, a starting PS concentration of 0.3 mM, and a pH value of 7. The industrial effluent was characterized, revealing a robust composition with initial concentrations of 150.74 mg/L for MB, 69.75 mg/L for TSS, 875.24 mg/L for dissolved organic matter (DOC), 54.72 ntu for turbidity, 263.75 mg/L for NH_3_, 1496.74 mg/L for TDS, and 78.63 mg/L for NO_3_^−^ (Table S3).

The removal of MB and DOC exhibited a consistent increase with prolonged exposure time, as evident in Table 5. At the culmination of a 120-min period, the (BC + light)/PS system attained the highest removal efficiencies of 98.31% for MB and 87.49% for DOC (Fig. 9b). Furthermore, the consumption of PS reached a rate of 84.7% (Fig. 9b) after 2 h, providing substantiation of PS activation by the (BC + light)/PS system. This activation resulted in the generation of radicals, playing a crucial role in the degradation process.

**Table 5:** RCs and REs% of MB dye and DOC in the raw and treated textile wastewater using the BC@(PS + light) system under the optimum conditions and different exposure times

Exposure time (min)	MB		DOC	
	RC (mg/L)	RE%	RC (mg/L)	RE%
0	150.74	–	246.99	–
20	121.02	19.72	212.18	14.09
40	93.88	37.72	169.6	31.33
60	69	54.23	135.27	45.23
80	41.85	72.24	93.32	62.22
100	2.27	84.56	63.34	74.36
120	2.55	98.31	36.55	85.2

The attainment of complete mineralization of real wastewater posed a challenge due to the intricate composition of the water matrix and the formation of organic intermediates. These intermediates led to an elevation in the concentration of DOC and impeded the accessibility of active sites, thereby diminishing the efficiency of reactive radicals generated during the treatment process. Additionally, competition arose between MB and other organic substances in the consumption of reactive radicals. Furthermore, the presence of inorganic ions in real wastewater (Table S3) had the potential to suppress the activity of reactive radicals, as highlighted by Samy et al. (2023a).

Despite these challenges, the (BC + light)/PS system exhibited promising outcomes in achieving a notable mineralization percentage. Additionally, the utilization of BC, known for its cost-effectiveness, enhances the feasibility and practicality of the proposed system for removing contaminants from industrial effluents. Furthermore, the suggested technique holds the potential to be incorporated as a tertiary unit in wastewater treatment plants, thereby opening avenues for the reuse of treated wastewater for irrigation purposes.

## Conclusion

The (BC + light)/PS system demonstrated superior MB removal efficiency at 83.36% compared to other systems tested in control experiments, prompting its selection for further experimentation. To optimize MB removal, operational conditions were systematically fine-tuned using response surface methodology. Under the optimal conditions, marked by an initial MB concentration of 8.5 mg/L, BC dosage of 0.15 g/L, initial PS concentration of 0.3 mM, and a reaction time of 120 min, the proposed system achieved an outstanding MB degradation rate of 99.02%. The obtained removal percentages for bromothymol blue dye (90.82%), paracetamol (81.88%), and chlorpyrifos (84.82%) underscored the notable efficiency of the treatment system in activating PS and degrading micropollutants. Moreover, the system exhibited remarkable stability and reusability over five consecutive cycles. Sulfate radicals were identified as predominant in MB degradation, surpassing other radical species in prevalence. In real textile wastewater, the proposed treatment system achieved a commendable removal rate of 98.31% for MB and 87.49% for DOC, highlighting its potential applicability in wastewater treatment plants and facilitating the reuse of treated wastewater for various purposes. The BC produced demonstrated cost-effectiveness, high porosity, elevated carbon content, and an abundance of oxygen-carbon functional groups, rendering it highly suitable for broader-scale applications.

Future research endeavors stemming from this study may include an exploration of additional emerging pollutants to augment the versatility and applicability of the proposed system. Moreover, a thorough assessment of the proposed system’s performance over prolonged durations and multiple cycles is imperative to gauge its durability and potential for real-world applications, additionally, a comprehensive cost analysis for the large-scale implementation of the proposed system to evaluate its viability for industrial applications, furthermore, an examination of the environmental impacts of the proposed system, encompassing the fate and potential toxicity of byproducts, for a comprehensive understanding of its implications, and lastly, the implementation of the suggested system in the treatment of authentic wastewater scenarios to furnish practical insights into its performance under realistic conditions.

## Supplementary Information

Below is the link to the electronic supplementary material.

Supplementary file1 (DOCX 1466 KB)

## Data Availability

All data generated or analyzed during this study are included in this published article [and its supplementary information files].

## References

[CR1] Adel A, Alalm MG, El-Etriby HK, Boffito DC (2020) Optimization and mechanism insights into the sulfamethazine degradation by bimetallic ZVI/Cu nanoparticles coupled with H2O2. J Environ Chem Eng 8:104341. 10.1016/J.JECE.2020.104341

[CR2] Ahmed N, Vione D, Rivoira L et al (2021) A review on the degradation of pollutants by Fenton-like systems based on zero-valent iron and persulfate: effects of reduction potentials, pH, and anions occurring in waste waters. Molecules 26. 10.3390/MOLECULES26154584 10.3390/molecules26154584 PMC8347750 34361737

[CR3] Al-Tohamy R, Ali SS, Li F et al (2022) A critical review on the treatment of dye-containing wastewater: ecotoxicological and health concerns of textile dyes and possible remediation approaches for environmental safety. Ecotoxicol Environ Saf 231:113160. 10.1016/J.ECOENV.2021.113160 35026583 10.1016/j.ecoenv.2021.113160

[CR4] Armah EK, Chetty M, Adedeji JA et al (2022) Biochar: production, application and the future. Biochar - Productive Technologies, Properties and Applications.10.5772/INTECHOPEN.105070

[CR5] Borowski E, Michałek S (2012) The effect of foliar feeding of potassium salts and urea in spinach on gas exchange, leaf yield and quality. Acta Agrobot 62:155–162. 10.5586/AA.2009.018

[CR6] Chowdhury ZZ, Ziaul Karim M, Ashraf MA, Khalid K (2016) Influence of carbonization temperature on physicochemical properties of biochar derived from slow pyrolysis of durian wood (Durio zibethinus) sawdust. BioResources 11:3356–3372. 10.15376/BIORES.11.2.3356-3372

[CR7] Cornejo OM, Piña FJ, Nava JL (2023a) Hybrid water treatment flow plant using hydrogen peroxide-based electro-activated persulfate and photoelectro-Fenton processes: the combustion of Reactive Orange 16 dye. J Ind Eng Chem 124:558–569. 10.1016/J.JIEC.2023.05.012

[CR8] Cornejo OM, Piña FJ, Nava JL (2023b) Hybrid water treatment flow plant using hydrogen peroxide-based electro-activated persulfate and photoelectro-Fenton processes: the combustion of Reactive Orange 16 dye. 10.1016/j.jiec.2023.05.012

[CR9] Darwish AS, Attia SK, Osman DI (2022) Accelerated activation of H2O2 and persulfate by Sm-doped ZnO@highly-defective layered yttria nanocomposite under visible-light irradiation for dyeing wastewater treatment: comprehensive dominance of oxygen vacancies in photocatalytic advanced oxidation processes. J Alloys Compd 925:166742. 10.1016/J.JALLCOM.2022.166742

[CR10] Deng X, Zhao Z, Wang C et al (2022) Insight into the nonradical mechanism of persulfate activation via visible-light for enhanced degradation of sulfonamides without catalyst. Appl Catal B 316:121653. 10.1016/J.APCATB.2022.121653

[CR11] Dzida K, Jarosz Z (2010) Effect of calcium carbonate and differentiated nitrogen fertilization upon the yield and chemical composition of spinach beet. Acta Scientiarum Polonorum Hortorum Cultus 09:201–210

[CR12] El-Bestawy EA, Gaber M, Shokry H, Samy M (2023) Effective degradation of atrazine by spinach-derived biochar via persulfate activation system: process optimization, mechanism, degradation pathway and application in real wastewater. Environ Res 229:115987. 10.1016/J.ENVRES.2023.115987 37116677 10.1016/j.envres.2023.115987

[CR13] Gogoi R, Dohling HM, Singh A et al (2022a) Visible light enhanced photosynthesis of C-C bonds using PdO/Pd@PEDOT nanocomposite. J Catal 414:109–124. 10.1016/J.JCAT.2022.08.027

[CR14] Gogoi R, Singh A, Moutam V et al (2022b) Revealing the unexplored effect of residual iron oxide on the photoreforming activities of polypyrrole nanostructures on plastic waste and photocatalytic pollutant degradation. J Environ Chem Eng 10:106649. 10.1016/J.JECE.2021.106649

[CR15] Habibi M, Habibi-Yangjeh A, Pouran SR et al (2022) Visible-light-triggered persulfate activation by CuCo2S4 modified ZnO photocatalyst for degradation of tetracycline hydrochloride. Colloids Surf A Physicochem Eng Asp 642:128640. 10.1016/J.COLSURFA.2022.128640

[CR16] Hu J, Zhang J, Wang Q et al (2019) Efficient degradation of tetracycline by ultraviolet-based activation of peroxymonosulfate and persulfate. Water Sci Technol 79:911–920. 10.2166/WST.2019.034 31025970 10.2166/wst.2019.034

[CR17] Ji L, Chen JW, Zheng ZG et al (2020) Excellent degradation performance of the Fe78Si11B9P2 metallic glass in azo dye treatment. J Phys Chem Solids 145:109546. 10.1016/J.JPCS.2020.109546

[CR18] Kumar V, Pandey N, Dharmadhikari S, Ghosh P (2020) Degradation of mixed dye via heterogeneous Fenton process: studies of calcination, toxicity evaluation, and kinetics. Water Environ Res 92:211–221. 10.1002/WER.1192 31373072 10.1002/wer.1192

[CR19] Kumi AG, Ibrahim MG, Fujii M, Nasr M (2020a) Synthesis of sludge-derived biochar modified with eggshell waste for monoethylene glycol removal from aqueous solutions. SN Appl Sci 2:1–12. 10.1007/S42452-020-03501-8/TABLES/1

[CR20] Kumi AG, Ibrahim MG, Fujii M, Nasr M (2020b) Synthesis of sludge-derived biochar modified with eggshell waste for monoethylene glycol removal from aqueous solutions. SN Appl Sci 2. 10.1007/S42452-020-03501-8

[CR21] Laura Bridgewater APHAAWWAWEF (2017) Standard methods for the examination of water and wastewater, 23rd edn. American Public Health Association

[CR22] Lee SK, Sheridan M, Mills A (2005) Novel UV-activated colorímetric oxygen indicator. Chem Mater 17:2744–2751. 10.1021/CM0403863/ASSET/IMAGES/MEDIUM/CM0403863N00001.GIF

[CR23] Li C, Dong Y, Yang J et al (2014) Modified nano-graphite/Fe3O4 composite as efficient adsorbent for the removal of methyl violet from aqueous solution. J Mol Liq 196:348–356. 10.1016/j.molliq.2014.04.010

[CR24] Li N, Wang Y, Cheng X et al (2022a) Influences and mechanisms of phosphate ions onto persulfate activation and organic degradation in water treatment: a review. Water Res 222:118896. 10.1016/J.WATRES.2022.118896 35914502 10.1016/j.watres.2022.118896

[CR25] Li X, Yao Y, Wang B (2022b) Incorporating Fe-O cluster in multivariate (MTV) metal–organic frameworks for promoting visible-light photo-Fenton degradation of micropollutants from water. Chem Eng J 446:137446. 10.1016/J.CEJ.2022.137446

[CR26] Li Y, Gupta R, Zhang Q, You S (2023b) Review of biochar production via crop residue pyrolysis: development and perspectives. Bioresour Technol 369:128423. 10.1016/J.BIORTECH.2022.128423 36462767 10.1016/j.biortech.2022.128423

[CR27] Li J, Wang Y, Ling H et al (2019) Significant enhancement of the visible light photocatalytic properties in 3d BiFeO3/graphene composites. Nanomaterials 9. 10.3390/nano9010065 10.3390/nano9010065 PMC6359105 30621245

[CR28] Li X, Cao H, Cao Y et al (2023a) Insights into the mechanism of persulfate activation with biochar composite loaded with Fe for 2,4-dinitrotoluene degradation. J Environ Manage 341. 10.1016/j.jenvman.2023.117955 10.1016/j.jenvman.2023.117955 37148765

[CR29] Liang S, Ziyu Z, Han J, Xiaoyan D (2021) Facile synthesis of magnetic mesoporous silica spheres for efficient removal of methylene blue via catalytic persulfate activation. Sep Purif Technol 256:1383–5866. 10.1016/j.seppur.2020.117801

[CR30] Liu T, Cui K, Li CX et al (2023) Efficient peroxymonosulfate activation by biochar-based nanohybrids for the degradation of pharmaceutical and personal care products in aquatic environments. Chemosphere 311. 10.1016/j.chemosphere.2022.137084 10.1016/j.chemosphere.2022.137084 36334754

[CR31] Luo J, Gao Y, Song T, Chen Y (2021) Activation of peroxymonosulfate by biochar and biochar-based materials for degrading refractory organics in water: a review. Water Sci Technol 83:2327–2344. 10.2166/WST.2021.147 34032613 10.2166/wst.2021.147

[CR32] Mensah K, Mahmoud H, Fujii M, Shokry H (2022) Novel nano-ferromagnetic activated graphene adsorbent extracted from waste for dye decolonization. Journal of Water Process Engineering 45. 10.1016/j.jwpe.2021.102512

[CR33] Miserli K, Kogola D, Paraschoudi I, Konstantinou I (2022) Activation of persulfate by biochar for the degradation of phenolic compounds in aqueous systems. Chem Eng J Adv 9:100201. 10.1016/J.CEJA.2021.100201

[CR34] Nie Y, Zhang Y, Nie X et al (2023) Colloidal iron species driven enhanced H2O2 decomposition into hydroxyl radicals for efficient removal of methylene blue from water. J Hazard Mater 448:130949. 10.1016/J.JHAZMAT.2023.130949 36860077 10.1016/j.jhazmat.2023.130949

[CR35] Niu L, Zhang G, Xian G et al (2021) Tetracycline degradation by persulfate activated with magnetic γ-Fe2O3/CeO2 catalyst: performance, activation mechanism and degradation pathway. Sep Purif Technol 259:118156. 10.1016/J.SEPPUR.2020.118156

[CR36] Nollet LML, De Gelder LSP (eds) (2013) Handbook of water analysis. CRC Press

[CR37] Ouyang D, Chen Y, Yan J et al (2019) Activation mechanism of peroxymonosulfate by biochar for catalytic degradation of 1,4-dioxane: Important role of biochar defect structures. Chem Eng J 370:614–624. 10.1016/J.CEJ.2019.03.235

[CR38] Pervez MN, Telegin FY, Cai Y et al (2019) Efficient degradation of mordant blue 9 using the Fenton-activated persulfate system. Water (Switzerland) 11. 10.3390/W11122532

[CR39] Powell JJ, McNaughton SA, Jugdaohsingh R et al (2005) A provisional database for the silicon content of foods in the United Kingdom. Br J Nutr 94:804–812. 10.1079/BJN20051542 16277785 10.1079/bjn20051542

[CR40] Saien J, Jafari F (2022) Methods of persulfate activation for the degradation of pollutants: fundamentals and influencing parameters. Persulfate-based Oxidation Processes in Environmental Remediation 1–59. 10.1039/9781839166334-00001

[CR41] Samy M, Ibrahim MG, Alalm MG, Fujii M (2020a) Modeling and optimization of photocatalytic degradation of methylene blue using lanthanum vanadate. Mater Sci Forum 1008:97–103. 10.4028/WWW.SCIENTIFIC.NET/MSF.1008.97

[CR42] Samy M, Ibrahim MG, Gar Alalm M et al (2020b) Innovative photocatalytic reactor for the degradation of chlorpyrifos using a coated composite of ZrV2O7 and graphene nano-platelets. Chem Eng J 395:124974. 10.1016/J.CEJ.2020.124974

[CR43] Samy M, Ibrahim MG, Gar Alalm M, Fujii M (2020c) MIL-53(Al)/ZnO coated plates with high photocatalytic activity for extended degradation of trimethoprim via novel photocatalytic reactor. Sep Purif Technol 249:117173. 10.1016/J.SEPPUR.2020.117173

[CR44] Samy M, Gar Alalm M, Fujii M, Ibrahim MG (2021) Doping of Ni in MIL-125(Ti) for enhanced photocatalytic degradation of carbofuran: reusability of coated plates and effect of different water matrices. J Water Process Eng 44:102449. 10.1016/J.JWPE.2021.102449

[CR45] Samy M, Elkady M, Kamal A et al (2022) Novel biosynthesis of graphene-supported zero-valent iron nanohybrid for efficient decolorization of acid and basic dyes. Sustainability (Switzerland) 14:14188. 10.3390/SU142114188/S1

[CR46] Samy M, Kumi AG, Salama E et al (2023a) Heterogeneous activation of persulfate by a novel nano-magnetite/ZnO/activated carbon nanohybrid for carbofuran degradation: Toxicity assessment, water matrices, degradation mechanism and radical and non-radical pathways. Process Saf Environ Prot 169:337–351. 10.1016/j.psep.2022.11.038

[CR47] Samy M, Mensah K, El-Fakharany EM et al (2023b) Green valorization of end-of-life toner powder to iron oxide-nanographene nanohybrid as a recyclable persulfate activator for degrading emerging micropollutants. Environ Res 223:115460. 10.1016/J.ENVRES.2023.115460 36775090 10.1016/j.envres.2023.115460

[CR48] Samy M, Mossad M, Kh El-Etriby H (2019) Synthesized nano titanium for Methylene Blue removal under various operational conditions.10.5004/dwt.2019.24510

[CR49] Seow YX, Tan YH, Mubarak NM et al (2022) A review on biochar production from different biomass wastes by recent carbonization technologies and its sustainable applications. J Environ Chem Eng 10:107017. 10.1016/J.JECE.2021.107017

[CR50] Seyyedbagheri H, Alizadeh R, Mirzayi B (2023) A novel heterostructure ZnO/PbBiO2Cl as a type-II photocatalyst for persulfate activation in tetracycline degradation under visible light. J Mol Liq 383:122067. 10.1016/J.MOLLIQ.2023.122067

[CR51] Shokri A (2018) Application of Sono–photo-Fenton process for degradation of phenol derivatives in petrochemical wastewater using full factorial design of experiment. Int J Ind Chem 9:295–303. 10.1007/S40090-018-0159-Y/FIGURES/6

[CR52] Song T, Li G, Hu R et al (2022) Degradation of antibiotics via UV-activated peroxodisulfate or peroxymonosulfate: a review. Catalysts 2022 12:1025. 10.3390/CATAL12091025

[CR53] Su S, Liu Y, He W et al (2019) A novel graphene oxide-carbon nanotubes anchored α-FeOOH hybrid activated persulfate system for enhanced degradation of Orange II. J Environ Sci 83:73–84. 10.1016/J.JES.2019.02.015 10.1016/j.jes.2019.02.015 31221389

[CR54] Teixeira YN, de Paula Filho FJ, Bacurau VP et al (2022) Removal of Methylene Blue from a synthetic effluent by ionic flocculation. Heliyon 8:e10868. 10.1016/J.HELIYON.2022.E10868 36262293 10.1016/j.heliyon.2022.e10868 PMC9573891

[CR55] Tolba A, Gar Alalm M, Elsamadony M et al (2019) Modeling and optimization of heterogeneous Fenton-like and photo-Fenton processes using reusable Fe3O4-MWCNTs. Process Saf Environ Prot 128:273–283. 10.1016/J.PSEP.2019.06.011

[CR56] Tran ML, Fu CC, Juang RS (2019) Effects of water matrix components on degradation efficiency and pathways of antibiotic metronidazole by UV/TiO2 photocatalysis. J Mol Liq 276:32–38. 10.1016/J.MOLLIQ.2018.11.155

[CR57] Wang J, Wang S (2018) Activation of persulfate (PS) and peroxymonosulfate (PMS) and application for the degradation of emerging contaminants. Chem Eng J 334:1502–1517. 10.1016/J.CEJ.2017.11.059

[CR58] Wang Q, Rao P, Li G et al (2020) Degradation of imidacloprid by UV-activated persulfate and peroxymonosulfate processes: kinetics, impact of key factors and degradation pathway. Ecotoxicol Environ Saf 187:109779. 10.1016/J.ECOENV.2019.109779 31639643 10.1016/j.ecoenv.2019.109779

[CR59] WHO (2022) Guidelines for drinking-water quality: fourth edition incorporating the first and second addenda. World Health Organization 1–614 35417116

[CR60] Xiao S, Cheng M, Zhong H et al (2020) Iron-mediated activation of persulfate and peroxymonosulfate in both homogeneous and heterogeneous ways: a review. Chem Eng J 384:123265. 10.1016/J.CEJ.2019.123265

[CR61] Xu L, Gao H, Shi Y, Zhao Y (2020) The heterogeneous volume-volatility relations in the exchange-traded fund market: evidence from China. Econ Model 85:400–408. 10.1016/J.ECONMOD.2019.11.019

[CR62] Xu R, Li M, Zhang Q (2022) Collaborative optimization for the performance of ZnO/biochar composites on persulfate activation through plant enrichment-pyrolysis method. Chem Eng J 429:132294. 10.1016/J.CEJ.2021.132294

[CR63] Yadav N, Garg VK, Chhillar AK, Rana JS (2021) Detection and remediation of pollutants to maintain ecosustainability employing nanotechnology: a review. Chemosphere 280 10.1016/j.chemosphere.2021.130792 34162093

[CR64] Yu Y, Guo H, Zhong Z et al (2022) Fe3O4 loaded on ball milling biochar enhanced bisphenol a removal by activating persulfate: performance and activating mechanism. J Environ Manag 319:115661. 10.1016/J.JENVMAN.2022.115661 10.1016/j.jenvman.2022.115661 35803072

[CR65] Zhang Y, Zhang BT, Teng Y et al (2021) Heterogeneous activation of persulfate by carbon nanofiber supported Fe3O4@carbon composites for efficient ibuprofen degradation. J Hazard Mater 401:123428. 10.1016/J.JHAZMAT.2020.123428 32659590 10.1016/j.jhazmat.2020.123428

[CR66] Zhang H, Mei Y, Zhu F et al (2022a) Efficient activation of persulfate by C@Fe3O4 in visible-light for tetracycline degradation. Chemosphere 306:135635. 10.1016/J.CHEMOSPHERE.2022.135635 35810856 10.1016/j.chemosphere.2022.135635

[CR67] Zhang Y, Yang Q, Liang J et al (2022b) Fe-glycerate microspheres as a heterogeneous catalyst to activate peroxymonosulfate for efficient degradation of methylene blue. J Phys Chem Solids 169:110893. 10.1016/J.JPCS.2022.110893

[CR68] Zhang X, Peng M, Zhang Q et al (2023) UV-photoaging behavior of polystyrene microplastics enhanced by thermally-activated persulfate. J Environ Chem Eng 11:110508. 10.1016/J.JECE.2023.110508

[CR69] Zhu Y, Wei J, Li J (2023) Biochar-activated persulfate oxidation of arsenic(III): nonnegligible roles of environmentally persistent free radicals. J Environ Chem Eng 111033. 10.1016/J.JECE.2023.111033

