# Supplementary material for: Effective degradation of synthetic micropollutants and real textile wastewater via a visible light-activated persulfate system using novel spinach leaf-derived biochar
Source: Environ Sci Pollut Res Int. 2024 Mar 11;31(17):25163–81. doi: 10.1007/s11356-024-32829-6 (PMC11636759; doi:10.1007/s11356-024-32829-6)
Supplement: Supplementary file 1 — Supplementary file1 (DOCX 1466 KB) [file 11356_2024_32829_MOESM1_ESM.docx]

**Effective degradation of synthetic micropollutants and real textile wastewater via a visible light-activated persulfate system using novel spinach leaves-derived biochar**

Mohamed Mohamed Gaber ^^[[1]](#footnote-1)^*^, Mahmoud Samy ^2^, Hassan Shokry ^3^

^1,3^ Environmental Engineering Department, Egypt-Japan University of Science and Technology (E-JUST), New Borg El‑Arab City 21934, Alexandria, Egypt

E-mail: [mohamed.gaber@ejust.edu.eg](mailto:mohamed.gaber@ejust.edu.eg)

E-mail: [hassan.shokry@ejust.edu.eg](mailto:hassan.shokry@ejust.edu.eg)

^2^ Department of Public Works Engineering, Faculty of Engineering, Mansoura University, Mansoura 35516, Egypt

E-mail: [msa203050@mans.edu.eg](mailto:msa203050@mans.edu.eg)

# Supplementary Figures


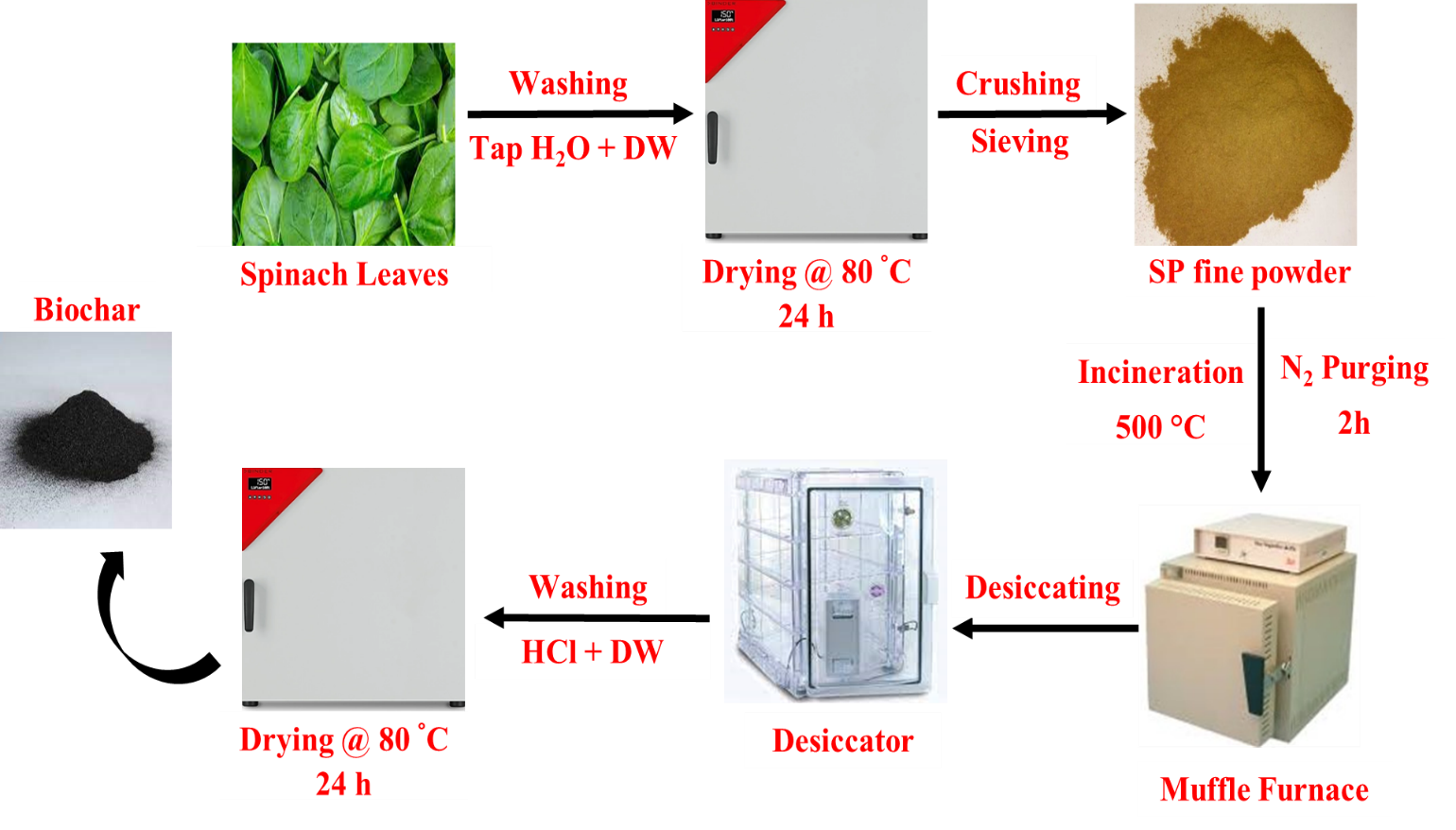


**Fig. S1**. Procedural schematic for biochar synthesis from spinach leaves.

**
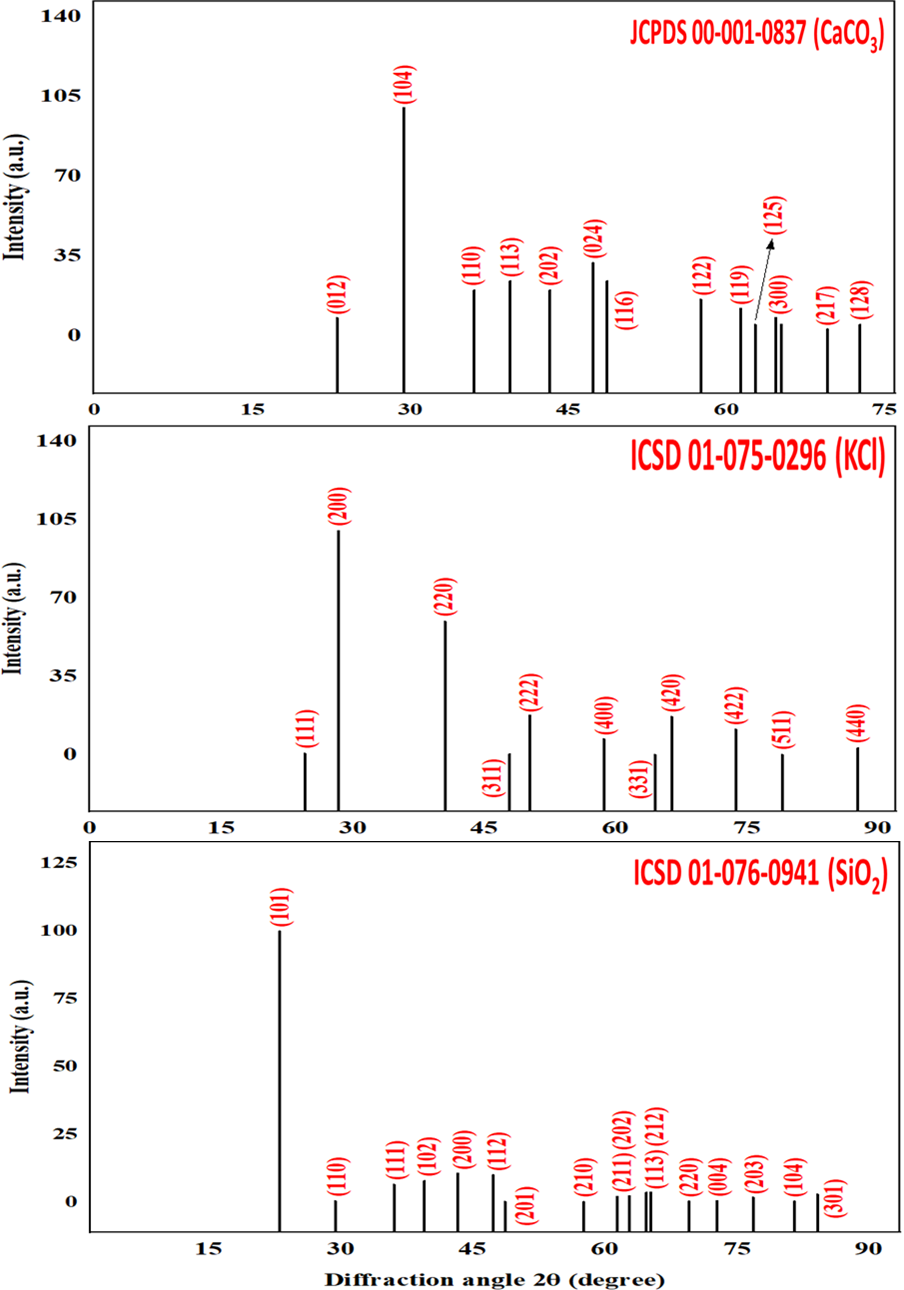
**

**Fig. S2.** XRD patterns of CaCO_3_, KCl, and SiO_2_ standard cards.


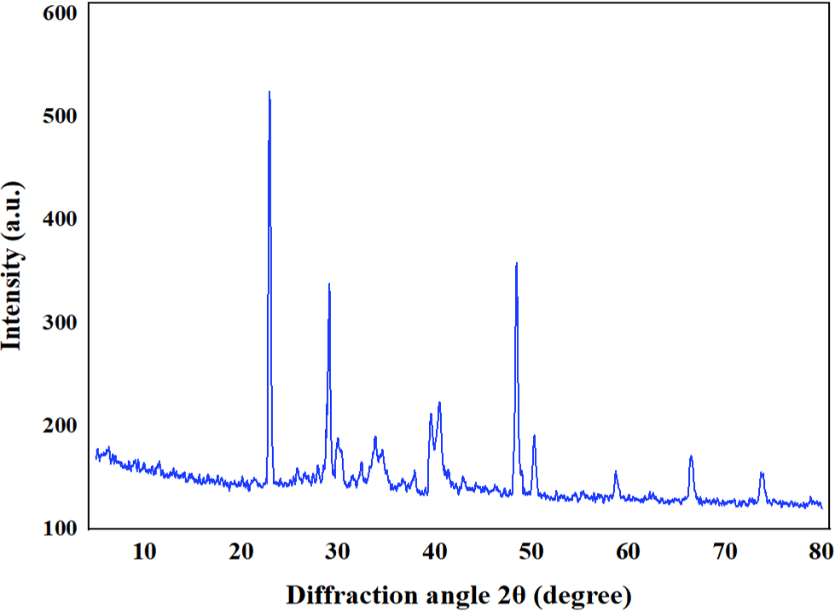

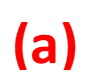

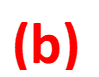

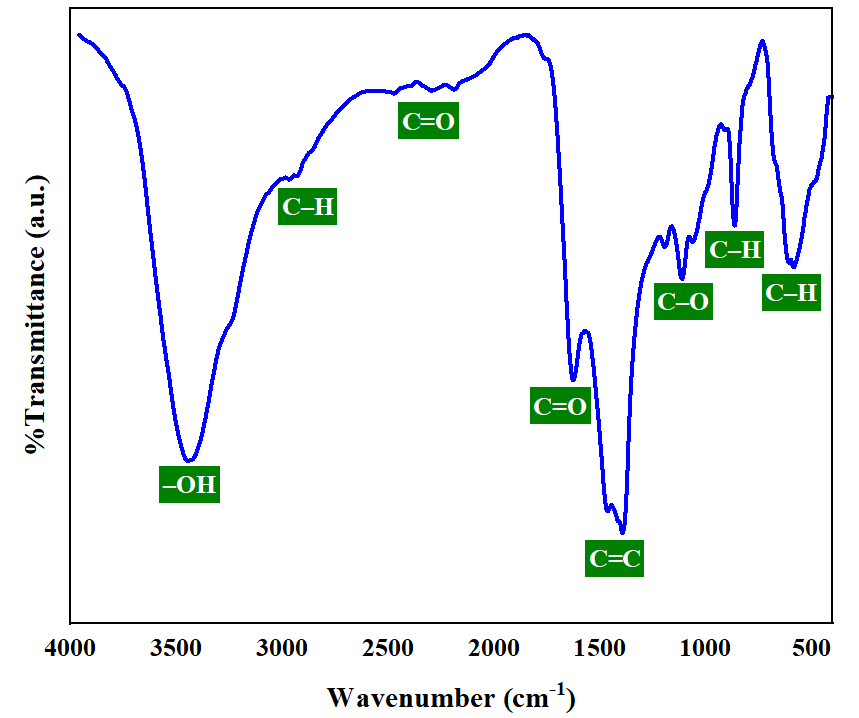


**Fig. S3.** (a) XRD and (b) FTIR of the synthesized biochar after the 5^th^ run (120 min/run).

**Fig. S4.** Leached iron residual concentrations over five successive runs using the BC@(PS + Light) system (conditions: initial MB concentration = 8.5 mg/L, BC dosage = 0.15 g/L, initial PS concentration = 0.3 mM, and run duration = 120 min).

**
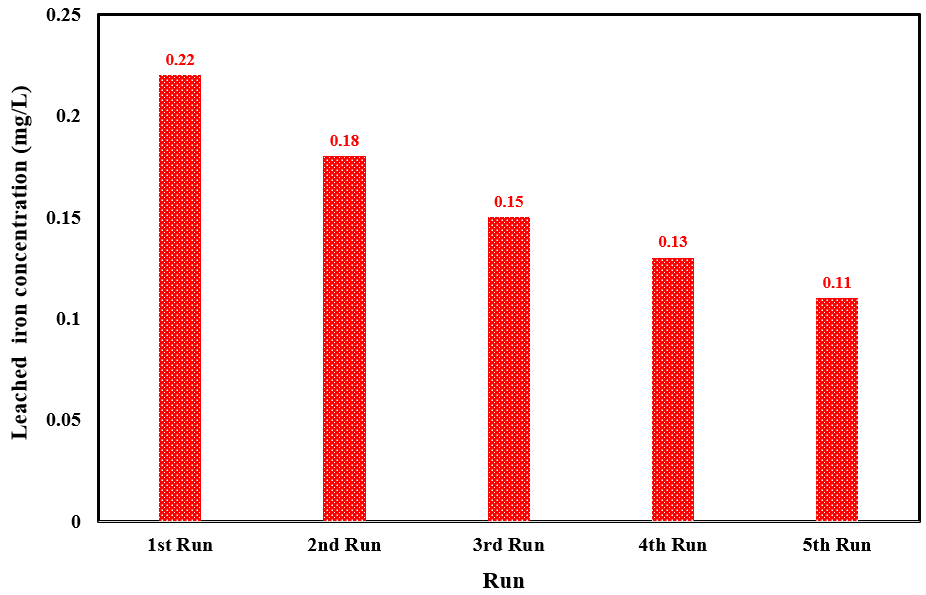
**

# Supplementary Tables

**Table S1** Equipment utilized for characterizing the synthesized biochar.

| Equipment | Model |
| --- | --- |
| FTIR spectroscopy | FTIR-8400S, Shimadzu, Japan |
|  |  |
| XRD analyzer | XRD 6000, Shimadzu, Japan |
|  |  |
| XRF analyzer | NEX CG, Rigaku, Japan |
|  |  |
| TEM, HRTEM, and SAED microscopy | JEM 2100F, Joel, Japan |
|  |  |
| EDS spectrometer | EDS spectrometer X-Max 80 mm2, Oxford, UK |

**Table S2** Instruments and techniques employed for analyzing the characteristics of raw and treated water.

| Parameter | Determination Method/ Equipment | References |
| --- | --- | --- |
| Methylene blue absorbance | UV/Vis. spectrophotometer (Evolution 350, Thermo Fisher Scientific, USA)@wavelength (λ) = 660 nm | (Samy et al. 2019) |
|  |  |  |
| Bromothymol blue absorbance | UV/Vis. spectrophotometer @ λ = 430 nm | (Hoag et al. 2009) |
|  |  |  |
| Paracetamol absorbance | UV/Vis. spectrophotometer @ λ = 244 nm | (Maneewong et al. 2022) |
|  |  |  |
| Chlorpyrifos absorbance | UV/Vis. spectrophotometer @ λ = 244 nm | (Raj and Kumar 2022) |
|  |  |  |
| Persulfate absorbance | Potassium iodide (KI) colorimetric method, using UV/Vis. spectrophotometer @ λ = 352 nm | (Liang et al. 2008) |
|  |  |  |
| Total organic carbon  (TOC) | TOC analyzer (TOC-L CPH/CPN, Shimazdu, Japan) | (Laura Bridgewater 2017) |
|  |  |  |
| Dissolved organic carbon  (DOC) | TOC analyzer for samples passed through 0.45-µm filter prior to analysis. | (Laura Bridgewater 2017) |
|  |  |  |
| UV absorbance @ 254 nm | UV/Vis. spectrophotometer @ λ = 254 nm |  |
|  |  |  |
| Specific UV absorbance (SUVA) | SUVA = [UV absorbance @ 254 / DOC] *100 | (Abd Manan et al. 2020) |
|  |  |  |
| Ammonia  (NH_3_) | SMWW 4500-NH_3_ B. (preliminary distillation Step) using steam distillation unit (Vapodest 30S, Gerhardt, Germany) + SMWW 4500-NH_3_ C. (Titrimetric Method) | (Laura Bridgewater, 2017) |
|  |  |  |
| Nitrates  (NO_3_^–^) | HACH #10020 (UV Spectrophotometric Screening Method) adapted from SMWW 4500-NO_3_^–^ B, using water quality spectrophotometer (DR1900, HACH, USA) | (Nollet and De Gelder 2013; Laura Bridgewater 2017) |
|  |  |  |
| Sulfates  (SO_4_^–2^) | HACH #10049 (USEPA SulfaVer 4 Method) adapted from SMWW, using water quality spectrophotometer | (Nollet and De Gelder 2013; Laura Bridgewater 2017) |
|  |  |  |
| Chlorides  (Cl^–^) | HACH #8225 (Silver Nitrate Buret Titration Method) adapted from SMWW 4500-Cl^–^ B. (Argentometric Method), using water quality spectrophotometer | (Nollet and De Gelder 2013; Laura Bridgewater 2017) |
|  |  |  |
| Potassium  (K) | Inductively coupled plasma mass spectrometer (ICP-MS) analyzer (7700x, Agilent, USA) | (Laura Bridgewater 2017) |
|  |  |  |
| Iron  (Fe) | Inductively coupled plasma mass spectrometer (ICP-MS) analyzer (7700x, Agilent, USA) | (Laura Bridgewater 2017) |
|  |  |  |
| pH | SMWW 4500-H^+^ B. (Electrometric Method) using digital multimeter (HQ440d, HACH, USA) and a pH probe (PHC301, HACH, USA) | (Laura Bridgewater 2017) |
|  |  |  |
| Electrical Conductivity (EC) | SMWW 2510-B. (Laboratory Method) using digital multimeter and a conductivity probe (CDC401, HACH, USA) | (Laura Bridgewater 2017) |
|  |  |  |
| Total Dissolved Solids (TDS) | SMWW 2540-C. (TDS Dried at 180°C) | (Laura Bridgewater 2017) |
|  |  |  |
| Total Suspended Solids (TSS) | SMWW 2540-D. (TSS Dried at 103–105°C) | (Laura Bridgewater 2017) |
|  |  |  |
| Turbidity  (ntu) | SMWW 2130-B. (Nephelometric Method) using turbidity meter (Turb 430T, WTW, USA) | (Laura Bridgewater 2017) |

**Table S3** Raw water analysis and characterization.

| Parameters | Unit | Textile WW | DW | TW | SW | LW | DRW |
| --- | --- | --- | --- | --- | --- | --- | --- |
| MB concentration | mg/L | 150.74 | 0 | 0 | 0 | 0 | 0 |
|  |  |  |  |  |  |  |  |
| DOC | mg/L | 875.24 | – | 49.76 | 65.43 | 80.08 | 106.06 |
|  |  |  |  |  |  |  |  |
| UV@254 nm | cm^–1^ | 2.46 | – | 0.98 | 1.52 | 1.75 | 2.72 |
|  |  |  |  |  |  |  |  |
| SUVA | L/mg.m | 0.28 | – | 1.98 | 2.33 | 2.18 | 2.57 |
|  |  |  |  |  |  |  |  |
| NH3 | mg/L | 263.75 | – | – | – | – | – |
|  |  |  |  |  |  |  |  |
| NO_3_^–^ | mg/L | 78.63 | 0 | 44.89 | 2.93 | 6.85 | 9.39 |
|  |  |  |  |  |  |  |  |
| SO_4_^–2^ | mg/L | – | 0 | 186.55 | 2.68 | 283.68 | 544.89 |
|  |  |  |  |  |  |  |  |
| Cl^–^ | mg/L | – | 0 | 222.87 | 17357.15 | 0.42 | 2.67 |
|  |  |  |  |  |  |  |  |
| pH | – | 7.83 | – | – | – | – | – |
|  |  |  |  |  |  |  |  |
| EC | µS/cm | 1108.71 | 0 | 0.78 | 45.84 | 0.46 | 1.89 |
|  |  |  |  |  |  |  |  |
| TDS | mg/L | 1496.74 | 6.86 | 314.42 | 3212.64 | 253.74 | 1237.57 |
|  |  |  |  |  |  |  |  |
| TSS | mg/L | 69.75 | – | – | – | – | – |
|  |  |  |  |  |  |  |  |
| Turbidity | ntu | 54.72 | 0 | 0.78 | 3.94 | 4.42 | 12.53 |

**Table S4** Functional groups of the synthesized biochar and their corresponding FTIR wavenumbers (cm^-1^), as well as a comparison with results reported in the literature.

| Functional groups | FTIR peaks (cm^–1^) of BC from spectrum |  | FTIR peaks from references | |
| --- | --- | --- | --- | --- |
|  |  |  | Peaks (cm^–1^) | References |
| C–H | 869.8  584.4 |  | 713 | (Xu et al. 2022) |
|  |  |  | 875 | (Xu et al. 2022) |
|  |  |  | 864, 580.6 | (El-Bestawy et al. 2023a) |
|  |  |  |  |  |
| C–O | 1195.8  1112.9 |  | 1035 | (Mensah et al. 2022) |
|  |  |  | 1051 | (Samy et al. 2023) |
|  |  |  | 1088 | (Xu et al. 2022) |
|  |  |  | 1109.87 | (Huo et al. 2020) |
|  |  |  |  |  |
| C=C | 1394.6 |  | 1388 | (El-Bestawy et al. 2023a) |
|  |  |  | 1458 | (El-Bestawy et al. 2023a) |
|  |  |  |  |  |
| C=O | 1627.8  2474.6  2293.2  2185.3 |  | 1624 | (El-Bestawy et al. 2023a) |
|  |  |  | 1630 | (Hasanpour et al. 2013) |
|  |  |  | 2344 | (Li et al. 2014) |
|  |  |  | 2356 | (Samy et al. 2023) |
|  |  |  |  |  |
| –OH | 3442.8 |  | 3412 | (Samy et al. 2023) |
|  |  |  | 3440 | (Xu et al. 2022) |
|  |  |  | 3500 | (Ahadpour Shal and Jafari 2014) |

**Table S5** XRD peak positions (2θ^◦^) and corresponding miller indices (hkl) identified in the synthesized biochar based on reference card data.

| Diffraction angles (2θ^◦^) of BC | Miller indices (hkl) from reference cards | | |
| --- | --- | --- | --- |
|  | JCPD 001-0837  (CaCO_3_) | ICSD 01-075-0296  (KCl) | ICSD 01-076-0941  (SiO_2_) |
| 22.9 | 012 | – | – |
|  |  |  |  |
| 27.88 | – | 200 | – |
|  |  |  |  |
| 29.1 | 104 | – | – |
|  |  |  |  |
| 29.96 | 104 | – | – |
|  |  |  |  |
| 36.66 | 110 | – | 112 |
|  |  |  |  |
| 39.62 | 113 | – | – |
|  |  |  |  |
| 40.48 | – | 220 | 210 |
|  |  |  |  |
| 42.88 | – | – | 211 |
|  |  |  |  |
| 47.26 | 024 | 311 | – |
|  |  |  |  |
| 48.44 | 116 | – | 212 |
|  |  |  |  |
| 50.28 | – | 222 | – |
|  |  |  |  |
| 58.7 | – | 400 | 310 |
|  |  |  |  |
| 66.46 | – | 420 | 223 |
|  |  |  |  |
| 73.7 | – | 422 | 322 |

**Table S6** Weight ratios of elements and oxides in the synthesized biochar.

| Elements’ ratios (wt%) | | |  | Oxides’ ratios (wt%) | | |
| --- | --- | --- | --- | --- | --- | --- |
| Element | Before treatment | After treatment |  | Oxide | Before treatment | After treatment |
| Mg | 1.36 | 1.27 |  | Na_2_O | 66.24 | 66.27 |
|  |  |  |  |  |  |  |
| Al | 0.119 | 0.109 |  | MgO | 4.79 | 4.63 |
|  |  |  |  |  |  |  |
| Si | 1.7 | 1.72 |  | K_2_O | 21.22 | 21.2 |
|  |  |  |  |  |  |  |
| P | 1.19 | 1.171 |  | CaO | 2.02 | 2.107 |
|  |  |  |  |  |  |  |
| S | 1.66 | 1.668 |  | MnO | 0.0183 | 0.0194 |
|  |  |  |  |  |  |  |
| Mn | 0.151 | 0.154 |  | Fe_2_O_3_ | 0.672 | 0.64 |
|  |  |  |  |  |  |  |
| Fe | 4.99 | 5 |  | Co_2_O_3_ | 0.0057 | 0.0076 |
|  |  |  |  |  |  |  |
| Co | 0.031 | 0.035 |  | NiO | 0.0016 | 0.0017 |
|  |  |  |  |  |  |  |
| Ni | 0.0188 | 0.0198 |  | CuO | 0.0068 | 0.0067 |
|  |  |  |  |  |  |  |
| Cu | 0.0604 | 0.0659 |  | ZnO | 0.0138 | 0.0143 |
|  |  |  |  |  |  |  |
| Zn | 0.118 | 0.18 |  | PtO_2_ | 0.001 | 0.001 |
|  |  |  |  |  |  |  |
| Pt | 0.0091 | 0.0084 |  | ZrO_2_ | 0.17 | 0.181 |
|  |  |  |  |  |  |  |
| Au | 0.0127 | 0.0179 |  | PbO | 0.0008 | 0.0013 |
|  |  |  |  |  |  |  |
| Na | 9.04 | 9.25 |  | SiO_2_ | 1.06 | 1.13 |
|  |  |  |  |  |  |  |
| K | 65 | 65.1 |  | Al_2_O_3_ | 1.28 | 1.29 |
|  |  |  |  |  |  |  |
| Ca | 13 | 12.8 |  | P_2_O_5_ | 1.44 | 1.39 |
|  |  |  |  |  |  |  |
| Zr | 1.54 | 1.431 |  | SO_3_ | 1.06 | 1.11 |

**Table S7** Comparison of persulfate activation and refractory dyes degradation: (BC+light)/PS system vs. other biochar-based systems from different sources"

| Biochar source | Pollutant | Degradation System | Operating Conditions | Removal efficiency | Reference |
| --- | --- | --- | --- | --- | --- |
| Tanned collagen fiber powder | Methylene blue  (MB) | BC/PS | [MB] = 20 mg/L, [BC] = 0.1 g/L, [PS] = 5 mM, T = 25 °C, pH = 7 | 92% | (Guo et al. 2022) |
|  |  |  |  |  |  |
| Peanut shell | Azo dye acid orange 7 (AO7) | BC/PS | [AO7] = 20 mg/L, [BC] = 0.25 g/L, [PS] = 30 mM, pH = 6 | 99% | (An et al. 2022) |
|  |  |  |  |  |  |
| Spinach remnants | MB | BC/PS | [MB] = 10 mg/L, [BC] = 1.88 g/L, [PS] = 7.7 mM, pH = 7, 120 min | 96.7% | (El-Bestawy et al. 2023b) |
|  |  |  |  |  |  |
| Phytolacca acinosa Roxb | MB | BC/PS | [MB] = 60 mg/L, [PI] = 1.0 mM, pH = 5.0, temperature = 25 ^◦^C | 3.9% | (Gong et al. 2024) |
|  |  |  |  |  |  |
| Poplar sawdust | AO7 | BC/PS | [AO7] = 20 mg/L, [BC] = 0.5 g/L, [PS] = 10 mM, pH = 6 | 99.6% | (Zhu et al. 2019) |
|  |  |  |  |  |  |
| Sewage sludge | AO7 | BC/PS | [AO7] = 0.06 mg/L, [BC] = 0.5 g/L, [PS] = 0.925 mM, pH = 5.22, 60 min | 59.9% | (Wang et al. 2020) |
|  |  |  |  |  |  |
| Spinach leaves | MB | BC/PS/visible light | [MB] = 8.5 mg/L, [BC] = 0.15 g/L, [PS] = 0.3 mM, pH = 7, 120 min | 99.02% | This study |

# References

Abd Manan TSB, Khan T, Wan Mohtar WHM, et al (2020) Dataset on specific UV absorbances (SUVA254) at stretch components of Perak River basin. Data Brief 30:105518. https://doi.org/10.1016/J.DIB.2020.105518

Ahadpour Shal A, Jafari A (2014) Study of structural and magnetic properties of superparamagnetic Fe 3 O 4 -ZnO core-shell nanoparticles. J Supercond Nov Magn 27:1531–1538. https://doi.org/10.1007/s10948-013-2469-9

An Q, Liu C, Deng S, et al (2022) Application of biochar activated persulfate in the treatment of typical azo pigment wastewater. J Environ Manage 324:116323. https://doi.org/10.1016/J.JENVMAN.2022.116323

El-Bestawy EA, Gaber M, Shokry H, Samy M (2023a) Effective degradation of atrazine by spinach-derived biochar via persulfate activation system: Process optimization, mechanism, degradation pathway and application in real wastewater. Environ Res 229:. https://doi.org/10.1016/j.envres.2023.115987

El-Bestawy EA, Gaber M, Shokry H, Samy M (2023b) Effective degradation of atrazine by spinach-derived biochar via persulfate activation system: Process optimization, mechanism, degradation pathway and application in real wastewater. Environ Res 229:115987. https://doi.org/10.1016/J.ENVRES.2023.115987

Gong J, Jiang H, Li X, et al (2024) Highly efficient activation of periodate by a manganese-modified biochar to rapidly degrade methylene blue. Environ Res 241:117657. https://doi.org/10.1016/J.ENVRES.2023.117657

Guo L, Zhao L, Tang Y, et al (2022) An iron–based biochar for persulfate activation with highly efficient and durable removal of refractory dyes. J Environ Chem Eng 10:106979. https://doi.org/10.1016/J.JECE.2021.106979

Hasanpour A, Niyaifar M, Asan M, Amighian J (2013) Synthesis and characterization of Fe3O4 and ZnO nanocomposites by the sol-gelmethod. J Magn Magn Mater 334:41–44. https://doi.org/10.1016/J.JMMM.2013.01.016

Hoag GE, Collins JB, Holcomb JL, et al (2009) Degradation of bromothymol blue by “greener” nano-scale zero-valent iron synthesized using tea polyphenols. J Mater Chem 19:8671–8677. https://doi.org/10.1039/b909148c

Huo X, Zhou P, Zhang J, et al (2020) N, S-Doped porous carbons for persulfate activation to remove tetracycline: Nonradical mechanism. J Hazard Mater 391:. https://doi.org/10.1016/J.JHAZMAT.2020.122055

Laura Bridgewater APHAAWWAWEF (2017) Standard Methods for the Examination of Water and Wastewater, 23rd edn. American Public Health Association

Li C, Dong Y, Yang J, et al (2014) Modified nano-graphite/Fe3O4 composite as efficient adsorbent for the removal of methyl violet from aqueous solution. J Mol Liq 196:348–356. https://doi.org/10.1016/j.molliq.2014.04.010

Liang C, Huang CF, Mohanty N, Kurakalva RM (2008) A rapid spectrophotometric determination of persulfate anion in ISCO. Chemosphere 73:1540–1543. https://doi.org/10.1016/j.chemosphere.2008.08.043

Maneewong Y, Chaemchuen S, Verpoort F, Klomkliang N (2022) Paracetamol removal from water using N-doped activated carbon derived from coconut shell: Kinetics, equilibrium, cost analysis, heat contributions, and molecular-level insight. Chemical Engineering Research and Design 185:163–175. https://doi.org/10.1016/J.CHERD.2022.07.007

Mensah K, Mahmoud H, Fujii M, Shokry H (2022) Novel nano-ferromagnetic activated graphene adsorbent extracted from waste for dye decolonization. Journal of Water Process Engineering 45:. https://doi.org/10.1016/j.jwpe.2021.102512

Nollet LML, De Gelder LSP (eds) (2013) Handbook of Water Analysis. CRC Press

Raj A, Kumar A (2022) Recent advances in assessment methods and mechanism of microbe-mediated chlorpyrifos remediation. Environ Res 214:114011. https://doi.org/10.1016/J.ENVRES.2022.114011

Samy M, Kumi AG, Salama E, et al (2023) Heterogeneous activation of persulfate by a novel nano-magnetite/ZnO/activated carbon nanohybrid for carbofuran degradation: Toxicity assessment, water matrices, degradation mechanism and radical and non-radical pathways. Process Safety and Environmental Protection 169:337–351. https://doi.org/10.1016/j.psep.2022.11.038

Samy M, Mossad M, Kh El-Etriby H (2019) Synthesized nano titanium for Methylene Blue removal under various operational conditions. https://doi.org/10.5004/dwt.2019.24510

Wang J, Shen M, Gong Q, et al (2020) One-step preparation of ZVI-sludge derived biochar without external source of iron and its application on persulfate activation. https://doi.org/10.1016/j.scitotenv.2020.136728

Xu R, Li M, Zhang Q (2022) Collaborative optimization for the performance of ZnO/biochar composites on persulfate activation through plant enrichment-pyrolysis method. Chemical Engineering Journal 429:. https://doi.org/10.1016/j.cej.2021.132294

Zhu K, Wang X, Chen D, et al (2019) Wood-based biochar as an excellent activator of peroxydisulfate for Acid Orange 7 decolorization. https://doi.org/10.1016/j.chemosphere.2019.05.087

1. Corresponding author:

   * E-mail: [mohamed.gaber@ejust.edu.eg](mailto:mohamed.gaber@ejust.edu.eg) (M.M. Gaber), Tel: +201023798513 [↑](#footnote-ref-1)
